# AutoMorph: Automated Retinal Vascular Morphology Quantification Via a Deep Learning Pipeline

**DOI:** 10.1167/tvst.11.7.12

**Published:** 2022-07-14

**Authors:** Yukun Zhou, Siegfried K. Wagner, Mark A. Chia, An Zhao, Peter Woodward-Court, Moucheng Xu, Robbert Struyven, Daniel C. Alexander, Pearse A. Keane

**Affiliations:** 1 Centre for Medical Image Computing, University College London, London, UK; 2 NIHR Biomedical Research Centre for Ophthalmology, Moorfields Eye Hospital NHS Foundation Trust and UCL Institute of Ophthalmology, London, UK; 3 Department of Computer Science, University College London, London, UK; 4 Department of Medical Physics and Biomedical Engineering, University College London, London, UK; 5 Institute of Health Informatics, University College London, London, UK

**Keywords:** retinal fundus photograph, vascular analysis, deep learning, oculomics, external validation

## Abstract

**Purpose:**

To externally validate a deep learning pipeline (AutoMorph) for automated analysis of retinal vascular morphology on fundus photographs. AutoMorph has been made publicly available, facilitating widespread research in ophthalmic and systemic diseases.

**Methods:**

AutoMorph consists of four functional modules: image preprocessing, image quality grading, anatomical segmentation (including binary vessel, artery/vein, and optic disc/cup segmentation), and vascular morphology feature measurement. Image quality grading and anatomical segmentation use the most recent deep learning techniques. We employ a model ensemble strategy to achieve robust results and analyze the prediction confidence to rectify false gradable cases in image quality grading. We externally validate the performance of each module on several independent publicly available datasets.

**Results:**

The EfficientNet-b4 architecture used in the image grading module achieves performance comparable to that of the state of the art for EyePACS-Q, with an *F*_1_-score of 0.86. The confidence analysis reduces the number of images incorrectly assessed as gradable by 76%. Binary vessel segmentation achieves an *F*_1_-score of 0.73 on AV-WIDE and 0.78 on DR HAGIS. Artery/vein scores are 0.66 on IOSTAR-AV, and disc segmentation achieves 0.94 in IDRID. Vascular morphology features measured from the AutoMorph segmentation map and expert annotation show good to excellent agreement.

**Conclusions:**

AutoMorph modules perform well even when external validation data show domain differences from training data (e.g., with different imaging devices). This fully automated pipeline can thus allow detailed, efficient, and comprehensive analysis of retinal vascular morphology on color fundus photographs.

**Translational Relevance:**

By making AutoMorph publicly available and open source, we hope to facilitate ophthalmic and systemic disease research, particularly in the emerging field of oculomics.

## Introduction

The widespread availability of rapid, non-invasive retinal imaging has been one of the most notable developments within ophthalmology in recent decades. The significance of the retinal vasculature for assessing ophthalmic disease is well known; however, there is also growing interest in its capacity to provide valuable insights into systemic disease, a field that has been termed “oculomics.”^1^^–^^4^ Narrowing of the retinal arteries is associated with hypertension and atherosclerosis,^5^^–^^8^ and dilation of the retinal veins is linked with diabetic retinopathy.^9^^–^^11^ Increased tortuosity of the retinal arteries is also associated with hypercholesterolemia and hypertension.^12^^–^^14^ Considering that manual vessel segmentation and feature extraction can be extremely time consuming, as well as poorly reproducible,^15^ there has been growing interest in the development of tools that can extract retinal vascular features in a fully automated manner.

In recent decades, a large body of technical work has focused on retinal vessel map segmentation. Performance has improved dramatically by employing a range of techniques, from unsupervised graph- and feature-based methods^16^^–^^20^ to supervised deep learning models.^21^ Despite this progress, the widespread use of these techniques in clinical research has been limited by a number of factors. First, technical papers^21^^–^^25^ often focus on performing a single function while ignoring upstream and downstream tasks, such as preprocessing^24^^,^^25^ and feature measurement.^21^^–^^23^ Second, existing techniques often perform poorly when applied to real-world clinical settings limited by poor generalizability outside of the environment in which they were developed.^26^^,^^27^

Although some software has been utilized for clinical research, most of it is only semi-automated, requiring human intervention for correcting vessel segmentation and artery/vein identification.^6^^,^^24^^,^^25^^,^^28^^,^^29^ This limits process efficiency and introduces subjective bias, potentially influencing the final outcomes. Further, most existing software has not integrated the crucial functions required for such a pipeline—namely, image cropping, quality assessment, segmentation, and vascular feature measurement. For example, poor-quality images in research cohorts often must be manually filtered by physicians, which generates a considerable workload. There is also the potential to improve the performance of underlying segmentation algorithms by employing the most recent advances in machine learning, thus enhancing the accuracy of vascular feature measurements.

In this study, we explored the feasibility of a deep learning pipeline providing automated analysis of retinal vascular morphology from color fundus photographs. We highlight three unique advantages of the proposed AutoMorph pipeline:

• AutoMorph consists of four functional modules, including (1) retinal image preprocessing; (2) image quality grading; (3) anatomical segmentation (binary vessel segmentation, artery/vein segmentation, and optic disc segmentation); and (4) morphological feature measurement.
• AutoMorph alleviates the need for physician intervention by addressing two key areas. First, we employ an ensemble technique with confidence analysis to reduce the number of ungradable images that are incorrectly classified as being gradable (false gradable images). Second, accurate binary vessel segmentation and artery/vein identification reduce the need for manual rectification.
• AutoMorph generates a diverse catalog of retinal feature measurements that previous work indicates has the potential to be used for the exploration of ocular biomarkers for systemic disease.

Perhaps most importantly, we made AutoMorph publicly available with a view to stimulating breakthroughs in the emerging field of oculomics.

## Methods

**Figure 1.**
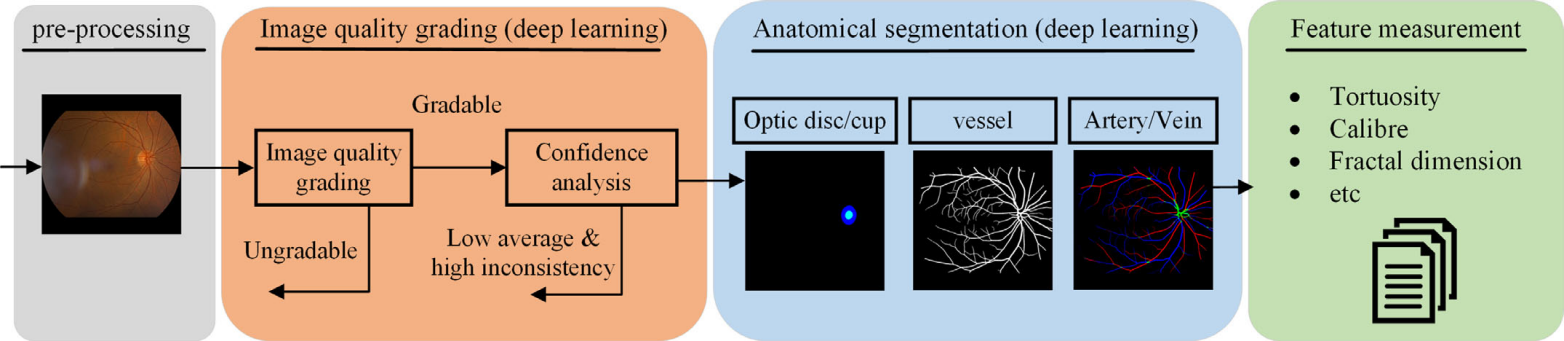
Diagram of the proposed AutoMorph pipeline. The input is color fundus photography, and the final output is the measured vascular morphology features. Image quality grading and anatomical segmentation modules use deep learning models. Confidence analysis decreases false gradable images in the image quality grading module.

The AutoMorph pipeline consists of four modules: (1) image preprocessing, (2) image quality grading, (3) anatomical segmentation, and (4) metric measurement (Fig. 1). Source code for this pipeline is available from https://github.com/rmaphoh/AutoMorph.

### Datasets

The datasets used for development and external validation of the deep learning models described in this work are summarized in Table 1 and Supplementary Material S1. For model training, we chose publicly available datasets that contain a large quantity of annotated images.^30^ Importantly, a diverse combination of public datasets was used in order to enhance external generalizability. Some image examples are shown in Supplementary Figure S1. To validate the models, we externally evaluated the performance of those trained models on datasets distinct from those on which they were trained (e.g., imaging devices, countries of origin, types of pathology). All of the datasets provide the retinal fundus photographs and the corresponding expert annotation. For image quality grading datasets (using EyePACS-Q as an example), two experts grade each image into three categories: good, usable, and reject quality, determined by image illumination, artifacts, and the diagnosability of the general eye diseases to the experts. For anatomical segmentation datasets, such as the Digital Retinal Images for Vessel Extraction (DRIVE) dataset for the binary vessel segmentation task, two experts annotate each pixel as vessel or background, thus generating a ground-truth map with the same size of the retinal fundus photographs, where a white color indicates vessel pixels and a black color the background. More details can be found in Supplementary Material S1.

**Table 1.** Characteristics of the Training and External Validation Data

Type of Data	Dataset Name	Country of Origin	Image Quantity^a^	Device (Manufacturer)
Image Quality Grading				
Training data	EyePACS-Q-train^30^^,^^31^	USA	12,543 (NR, more than 99%)	A variety of imaging devices, including DRS (CenterVue, Padova, Italy); iCam (Optovue, Fremont, CA); CR1/DGi/CR2 (Canon, Tokyo, Japan); Topcon NW 8 (Topcon, Tokyo, Japan)
Internal validation data	EyePACS-Q-test^30^^,^^31^	USA	16,249 (NR, more than 99%)	—
External validation data	DDR test^32^	China	4,105 (100%)	42 types of fundus cameras, mainly Topcon D7000, Topcon TRC NW48, D5200 (Nikon, Tokyo, Japan), and Canon CR 2 cameras

Binary Vessel Segmentation				
Training data	DRIVE^33^	Netherlands	40 (100%)	CR5 non-mydriatic 3CCD camera (Canon)
	STARE^34^	USA	20 (100%)	TRV-50 fundus camera (Topcon)
	CHASEDB1^35^	UK	28 (0%)	NM-200D handheld fundus camera (Nidek, Aichi, Japan)
	HRF^36^	Germany and Czech Republic	45 (100%)	CF-60UVi camera (Canon)
	IOSTAR^37^	Netherlands and China	30 (53.3%)	EasyScan camera (i-Optics, Rijswijk, Netherlands)
	LES-AV^38^	NR	22 (0%)	Visucam Pro NM fundus camera (Carl Zeiss Meditec, Jena, Germany)
External validation data^b^	AV-WIDE^19^^,^^39^	USA	30 (100%)	200Tx Ultra-widefield Imaging Device (Optos, Dunfermline, UK)
	DR HAGIS^40^	UK	39 (100%)	TRC-NW6s (Topcon), TRC-NW8 (Topcon), or CR-DGi fundus camera (Canon)

Artery/Vein Segmentation				
Training data	DRIVE-AV^33^^,^^41^	Netherlands	40 (100%)	CR5 non-mydriatic 3CCD camera (Canon)
	HRF-AV^36^^,^^42^	Germany and Czech Republic	45 (100%)	CF-60UVi camera (Canon)
	LES-AV^38^	Nauru	22 (9%)	Visucam Pro NM fundus camera (Zeiss)
External validation data	IOSTAR-AV^37^^,^^43^	Netherlands and China	30 (53.3%)	EasyScan camera (i-Optics)

Optic Disc Segmentation				
Training data	REFUGE^44^	China	800 (100%)	Visucam 500 fundus camera (Zeiss) and CR-2 camera (Canon)
	GAMMA^45^^,^^46^	China	100 (100%)	—
External validation data^c^	IDRID^47^	India	81 (100%)	VX-10α digital fundus camera (Kowa, Las Vegas, NV)

External validation data are unseen for model training and were purely used to evaluate the trained model performance on out-of-distribution data with different countries of origin and imaging devices. EyePACS-Q is a subset of EyePACS with image quality grading annotation. NR, not reported.

a Image quantity indicates the image number used in this work and the parentheses show the proportion of macula-centered images.

b Although we have evaluated the binary vessel segmentation model on the ultra-widefield retinal fundus dataset AV-WIDE, we recommend using AutoMorph on retinal fundus photographs with a 25° to 60° FOV, as all of the deep learning models are trained using images with FOV equals to 25° to 60°, and the preprocessing step is tailored for images with this FOV.

c Evaluated on disc due to no cup annotation.

### Modules

#### Image Preprocessing

Retinal fundus photographs often contain superfluous background, resulting in dimensions that deviate from a geometric square. To account for this, we employed a technique that combines thresholding, morphological image operations, and cropping^31^ to remove the background so that the resulting image conforms to a geometric square (examples are shown in Supplementary Fig. S2).

#### Image Quality Grading

To filter out ungradable images that often fail in segmentation and measurement modules, AutoMorph incorporates a classification model to identify ungradable images. The model classifies each image as good, usable, or reject quality. In our study, good and usable images were considered to be gradable; however, this decision may be modified in scenarios with sufficient data to include only good-quality images. We employed EfficientNet-B4^48^ as the model architecture and performed transfer learning on EyePACS-Q. Further details are outlined in Supplementary Material S2 and Supplementary Figure S3.

#### Anatomical Segmentation

Vascular structure is thin and elusive especially against low-contrast backgrounds. To enhance binary vessel segmentation performance, AutoMorph uses an adversarial segmentation network.^23^ Six public datasets were used for model training (Table 1). Accurate artery/vein segmentation is a long-standing challenge. To address this, we employed an information fusion network^22^ tailored for artery/vein segmentation. Three datasets were used for training. Parapapillary atrophic changes, which can be a hallmark of myopia or glaucoma, can cause large errors in disc localization and segmentation. To counter this, AutoMorph employs a coarse-to-fine deep learning network,^49^ which achieved first place for disc segmentation in the MICCAI 2021 GAMMA challenge.^45^^,^^46^ Two public datasets were utilized in model training. Further detailed information is provided in Supplementary Material S3.

#### Vascular Morphology Feature Measurement

**Figure 2.**
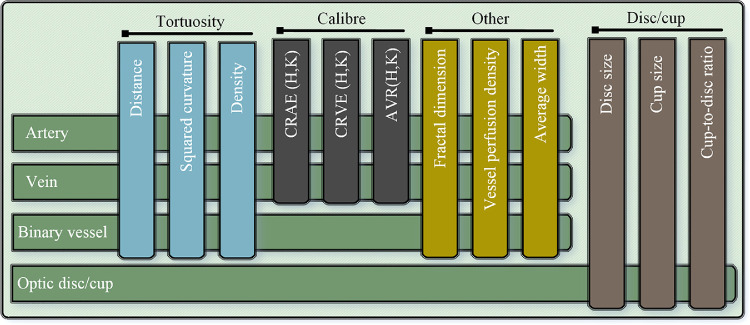
Features measured by AutoMorph, including tortuosity, vessel caliber, disc-to-cup ratio, and others. For each image, the optic disc/cup information is measured, including the height and width, as well as cup-to-disc ratio. For binary vessels, the tortuosity, fractal dimension, vessel density, and average width are measured. In addition to these features, arteries/veins are also used for measuring the caliber features CRAE, CRVE, and AVR by Hubbard and Knudtson methods.

AutoMorph measures a series of clinically relevant vascular features, as summarized in Figure 2 (comprehensive list in Supplementary Fig. S13). Three different calculation methods for vessel tortuosity are provided, including distance measurement tortuosity, squared curvature tortuosity,^50^ and tortuosity density.^51^ The fractal dimension value (Minkowski–Bouligand dimension)^52^ provides a measurement of vessel complexity. The vessel density indicates the ratio between the area of vessels to the whole image. For vessel caliber, AutoMorph calculates the central retinal arteriolar equivalent (CRAE) and central retinal venular equivalent (CRVE), as well as the arteriolar–venular ratio (AVR).^53^^–^^55^ AutoMorph measures the features in standard regions, including Zone B (the annulus 0.5–1 optic disc diameter from the disc margin) and Zone C (the annulus 0.5–2 optic disc diameter from the disc margin).^29^ Considering that Zone B and Zone C of macular-centered images may be out of the circular fundus, the features for the whole image are also measured.

### Ensemble and Confidence Analysis

**Figure 3.**
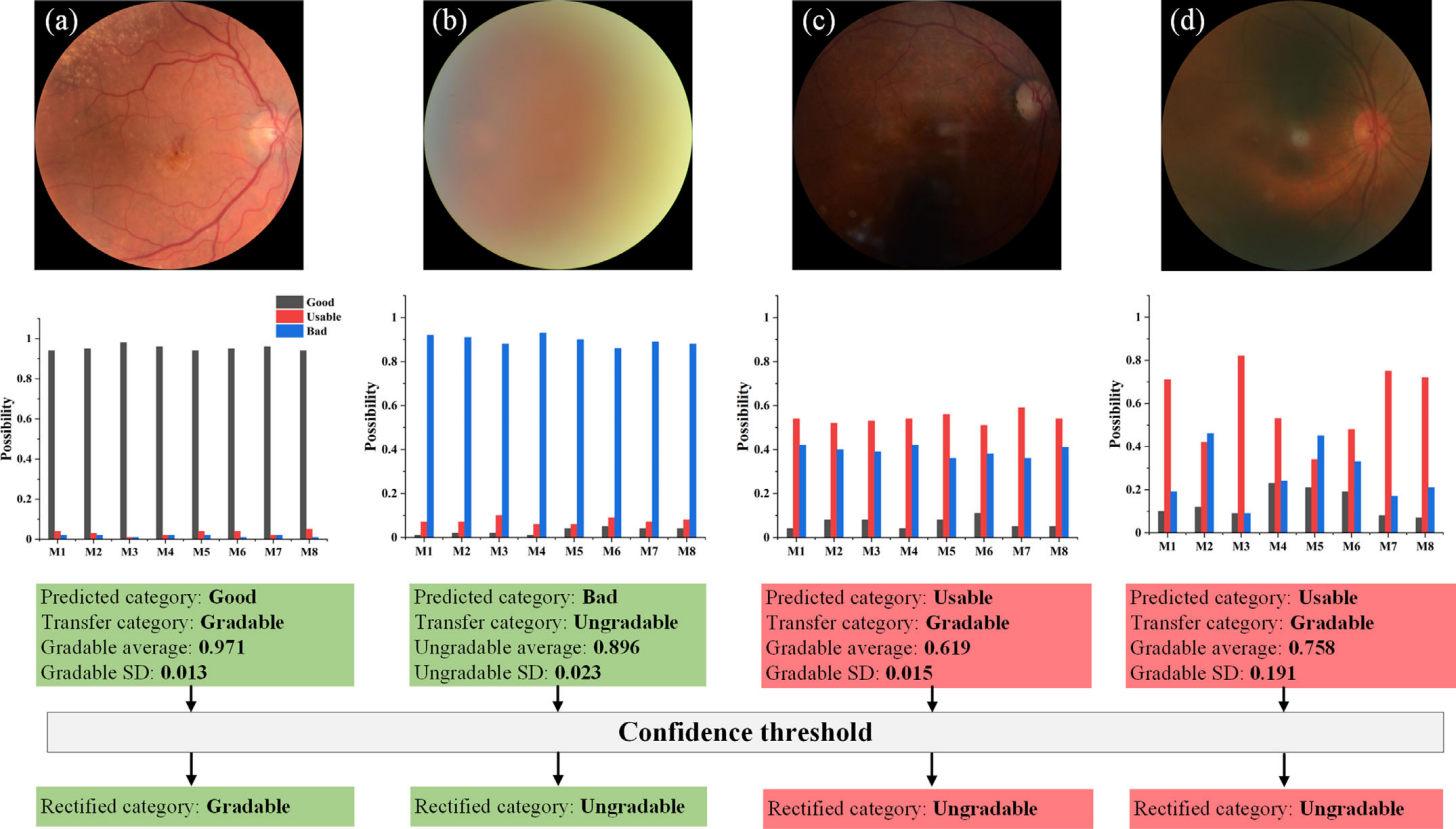
Confidence analysis for image quality grading. M1 to M8 represent the eight ensemble models. For each image, the predicted category is transferred as gradable or ungradable (good and usable are as gradable, reject as ungradable). The average probability and SD are calculated for the predicted category. (a, b) Two image cases with high confidence in prediction. The case shown in (c) is classified as gradable quality with low average probability of 0.619, and the case in (d) has a high SD of 0.191, which are defined as low-confidence images in our work. Although (c) and (d) are preliminarily classified as gradable, the final classification is rectified as ungradable with the confidence threshold.

In model training, 80% of the training data is used for model training and 20% is used to tune the training hyperparameters, such as scheduling the learning rate. In retinal image grading, we ensemble the output from eight trained models with different subsets of training data, as it generally gives a more robust result.^56^ Moreover, the average value and standard deviation (SD) of the eight possibilities are calculated for confidence analysis. Average probability indicates the average confidence of prediction. Low average cases are prone to false predictions, such as Figure 3c. Meanwhile, SD represents the inconsistency between models. High inconsistency likely corresponds to a false prediction, as shown in Figure 3d. The images with either low average probability or high SD are automatically recognized as low-confidence images and rectified as ungradable. False gradable images can fail the anatomical segmentation module, thus generating a large error in vascular feature measurement. The confidence analysis economizes physician intervention and increases the reliability of AutoMorph by filtering these potential errors. To our knowledge, this is the first report of a confidence analysis combined with the model ensemble integrated within the vessel analysis pipeline. An average threshold corresponds to a change of operating point and SD threshold involved in uncertainty theory. In this work, we set an average threshold of 0.75 and a SD threshold of 0.1 to filter out false gradable images. Specifically, the average probability lower than 0.75 or SD larger than 0.1 were rectified as ungradable images. The rationale for selecting these threshold values is based on the probability distribution histogram on tuning data. More details are described in Supplementary Material S2 and Supplementary Figure S4.

### Statistical Analyses and Compared Methods

For deep learning functional modules, the well-established expert annotation is used as a reference standard to quantitatively evaluate the module performance. We calculated sensitivity, specificity, positive predictive value (precision), accuracy, area under the receiver operating characteristic (AUC-ROC) curve, *F*_1_-score, and intersection of union (IoU) metrics to verify the model performance. These metric definitions are

$$\begin{array}{c} Accuracy=\frac{TP+TN}{TP+FP+TN+FN} \end{array}$$

$$\begin{array}{c} Sensitivity=\frac{TP}{TP+FN} \end{array}$$

$$\begin{array}{c} Specificity=\frac{TN}{TN+FP} \end{array}$$

$$\begin{array}{c} Precision=\frac{TP}{TP+FP} \end{array}$$

$$\begin{array}{c} F1=\frac{2\;\times Sensitivity\times Precision}{Sensitivity\;+\;Precision} \end{array}$$

where *TP*, *TN*, *FP*, and *FN* indicate true positive, true negative, false positive, and false negative, respectively. AUC-ROC curve is a performance measurement for classification problems at various threshold settings; it tells how much the model is capable of distinguishing between classes. In segmentation tasks, IoU measures the overlap degree between ground-truth maps and segmentation maps. Following the same setting,^31^^,^^39^^,^^57^^–^^59^ we set the ungradable images as the positive class in image quality grading. The probability of the ungradable category equals that of reject quality, and the probability of the gradable category is the sum of good quality and usable quality. As introduced in the discussion on confidence analysis, we used a mean value of 0.75 and SD of 0.1 as thresholds to obtain the final rectified gradable and ungradable categories. For binary vessel segmentation, each pixel of the retinal fundus photograph corresponds to a binary classification task. The vessel pixel is positive class and the background pixel is negative. The probability range for each pixel is from 0 to 1, where a larger value indicates a higher probability of being a vessel pixel. We thresholded the segmentation map with 0.5, which is a standard threshold for binary medical image segmentation tasks. Optic disc segmentation is similar to binary vessel segmentation, but the difference is that the positive class is the optic disc pixel. For artery/vein segmentation, each pixel has a four-class probability of artery, vein, uncertain pixel, and background. Following standard settings for multiclass segmentation tasks, the category with the largest probability across the four classes is the thresholded pixel category. More information is listed in Supplementary Material S3.

We conducted the quantitative comparison to other competitive methods to characterize the generalizability of AutoMorph using external validation. We used internal validation results from other published work to provide a benchmark for a well-performing model. These methods used a reasonable proportion of data for model training and the remainder for internal validation (e.g., fivefold validation that means 80% of images are used for training and tuning and 20% are used for validating the trained model), and claimed that they have achieved state-of-the-art performance. As introduced in Table 1, the models of AutoMorph are trained on several public datasets and externally validated on separate datasets, whereas the compared methods^39^^,^^57^^–^^59^ are trained in the same domain data as the validation data but with fewer training images. The goal of the comparison was not to prove the technical strengths of AutoMorph over recent methods, as this has already been verified in previously published work.^22^^,^^23^^,^^47^^,^^48^ Rather, we aimed to demonstrate that, due to the diversity of its training data, AutoMorph performs well on external datasets, even when these datasets include pathology and show large domain differences from the training data. Additionally, to demonstrate the technical superiority of this method, we have provided the internal validation of AutoMorph in Supplementary Table S1.

Considering that we employ standard formulas^29^^,^^50^^–^^52^ to measure vascular morphology features, the measurement error only comes from inaccuracy of anatomical segmentation. In order to evaluate measurement error that occurs as a result of vessel segmentation, we respectively measure the vascular features based on AutoMorph segmentation and expert vessel annotation, and then we draw Bland–Altman plots. Following the same evaluation,^3^^,^^60^ intraclass correlation coefficients (ICCs) are calculated to quantitatively show agreement. Additionally, the boxplots of differences between the vascular features from AutoMorph segmentation and expert annotation are shown in Supplementary Figures S9–S11.

## Results

Results for external validation of AutoMorph are summarized in Table 2.

**Table 2.** Validation of Functional Modules and Comparison With Other Methods

	Image Quality Grading				Artery/Vein Segmentation	
	EyePACS-Q Test		DDR Test		IOSTAR-AV	
	AutoMorph (Internal)	Comparison^31^ (Internal)	AutoMorph (External)	Comparison^a^ (Internal)	AutoMorph (External)	Comparison^58^ (Internal)
Sensitivity	0.85	0.85	1	0.93	0.64	0.79
Specificity	0.93	NR	0.89	0.97	0.98	0.76
Precision	0.87	0.87	0.6	0.73	0.68	NR
Accuracy	0.92	0.92	0.91	0.99	0.96	0.78
AUC-ROC	0.97	NR	0.99	0.99	0.95	NR
*F*_1_-score	0.86	0.86	0.75	0.82	0.66	NR
IoU	—	—	—	—	0.53	NR
	Binary Vessel Segmentation				Optic Disc	
	Ultra-widefield: AV-WIDE		Standard Field: DR HAGIS		IDRID	
	AutoMorph (External)	Comparison^39^ (Internal)	AutoMorph (External)	Comparison^57^ (Internal)	AutoMorph (External)	Comparison^59^ (Internal)

Sensitivity	0.71	0.78	0.84	0.67	0.9	0.9
Specificity	0.98	NR	0.98	0.98	0.95	NR
Precision	0.75	0.82	0.73	NR	0.94	NR
Accuracy	0.96	0.97	0.97	0.97	0.99	0.99
AUC-ROC	0.96	NR	0.98	NR	0.95	NR
*F*_1_-score	0.73	0.8	0.78	0.71	0.94	NR
IoU	0.57	NR	0.64	NR	0.91	0.85

“Internal” indicates that the validation and training data are from the same dataset but isolated. “External” means that validation data are from external datasets. The comparisons are with competitive methods of image quality grading,^31^ binary vessel segmentation,^39^^,^^57^ artery/vein segmentation,^58^ and optic disc segmentation.^59^ NR, not reported.

^a^Due to no comparison method on the DDR test, we compared AutoMorph (external) to the same architecture, EfficientNet-b4, that is trained with DDR train data (internal).

### Image Quality Grading

**Figure 4.**
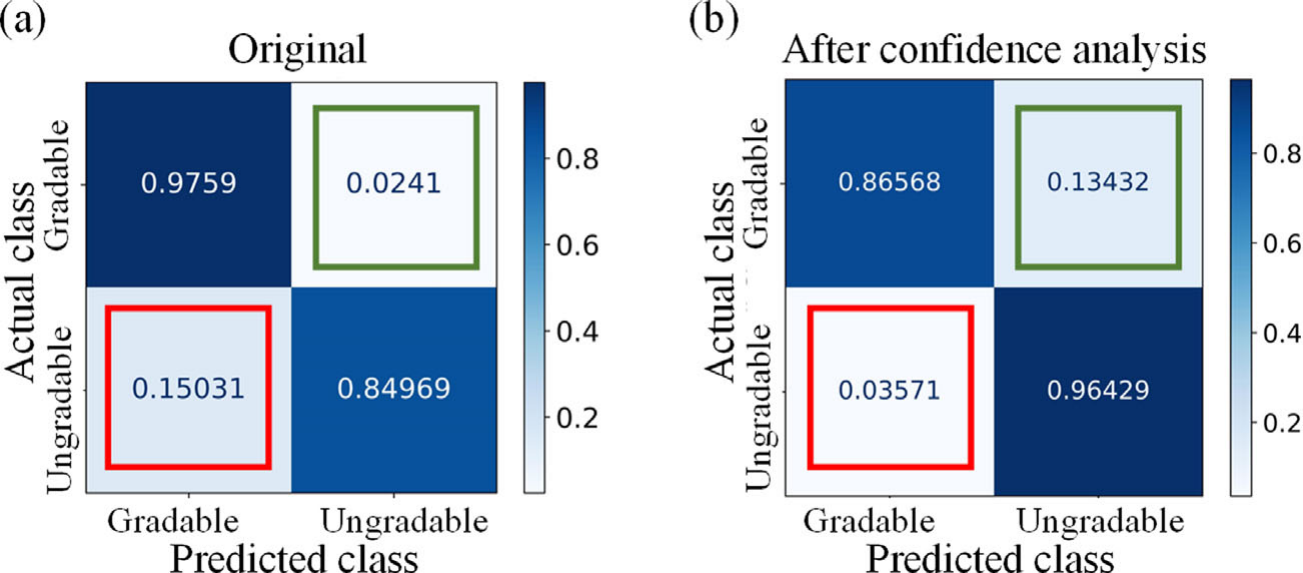
The confusion matrix of the grading results on EyePACS-Q test data. (a) The results before confidence thresholding; (b) the results after thresholding. The value is normalized in rows. The diagonal includes the correct classification ratio. The *red box* indicates false gradable (i.e., ungradable images are wrongly classified as gradable), and the *green box* shows the percentage of false ungradable (i.e., gradable images are wrongly categorized as ungradable). The false gradable of (b) is reduced by 76.2% compared with that of (a), but the false ungradable increases in (b).

The internal validation is on EyePACS-Q test data. For fair comparison,^31^ we evaluated the image quality grading performance of categorizing good, usable, and reject quality. The quantitative results are listed in Table 2. The classification *F*_1_-score achieved 0.86, on par with the state-of-the-art method with a *F*_1_-score of 0.86.^31^ The prediction was transferred to gradable (good and usable quality) and ungradable (reject quality), and the resulting confusion matrix of validation on the EyePACS-Q test is shown in Figure 4. We learned that confidence thresholding brings a trade-off in performance metrics, suppressing false gradable ratio but simultaneously increasing false negative. False gradable images are prone to fail the anatomical segmentation module and generate large errors and outliers in vascular feature measurement. Although this thresholding filters out some adequate quality images, it maintains the reliability of AutoMorph.

The external validation is on the general-purpose diabetic retinopathy dataset (DDR) test data. As DDR includes only two categories in image quality annotation (gradable and ungradable), we first transferred the AutoMorph prediction of good and usable quality as gradable and reject quality as ungradable and then evaluated the quantitative results. Although the difference in the annotation might underestimate the AutoMorph image quality grading capability, the performance was satisfactory compared to the internal group, as shown in Table 2. The confusion matrix and AUC-ROC curve are shown in Supplementary Figure S5. All ungradable images were correctly identified, which is significant with regard to the reliability of AutoMorph.

### Anatomical Segmentation

**Figure 5.**
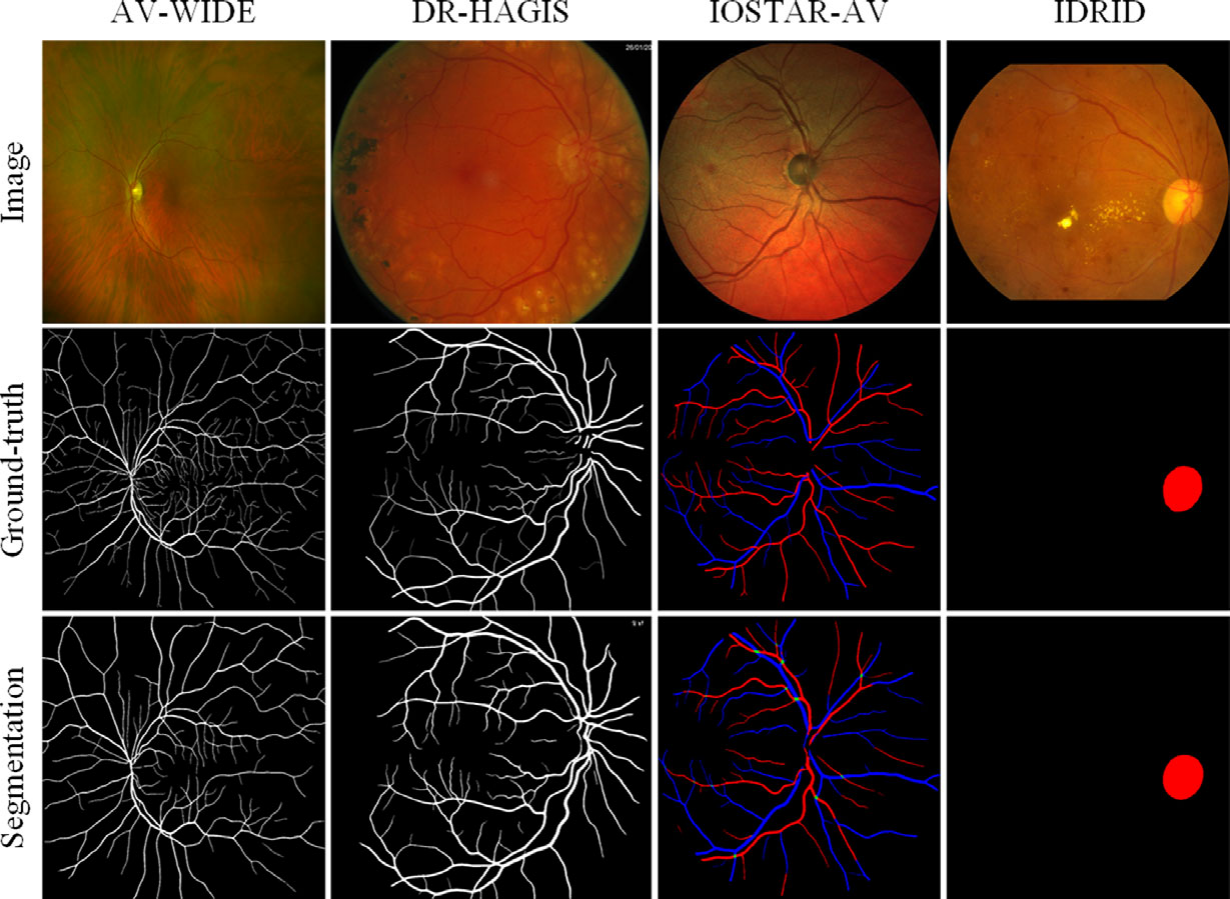
Visualization results of anatomical segmentation, including binary vessel (first two columns), artery/vein (third column), and optic disc (final column).

Visualization results are presented in Figure 5, and quantitative results are listed in Table 2. For binary vessel segmentation, the two public datasets AV-WIDE and the diabetic retinopathy, hypertension, age-related macular degeneration, and glaucoma image set (DR HAGIS) are employed in model validation. The binary vessel segmentation model works comparably to SOTA performance on the fundus photography data (DR HAGIS) and moderately so on ultra-widefield data (AV-WIDE). For artery/vein segmentation, the performance is validated on the IOSTAR-AV dataset. Compared with the most recent method,^58^ AutoMorph achieves lower sensitivity but much higher specificity. The visualization results of two challenging cases from Moorfields Eye Hospital and the Online Retinal Fundus Image Dataset for Glaucoma Analysis and Research (ORIGA) are shown in Supplementary Figure S6. For optic disc segmentation, we validated the performance on the dataset Indian Diabetic Retinopathy Image Dataset (IDRID). The performance is on the par with the compared method,^59^ and the *F*_1_-score is slightly higher. Although pathology disturbs, the segmentation disc shows robustness.

### Vascular Feature Measurement

**Figure 6.**
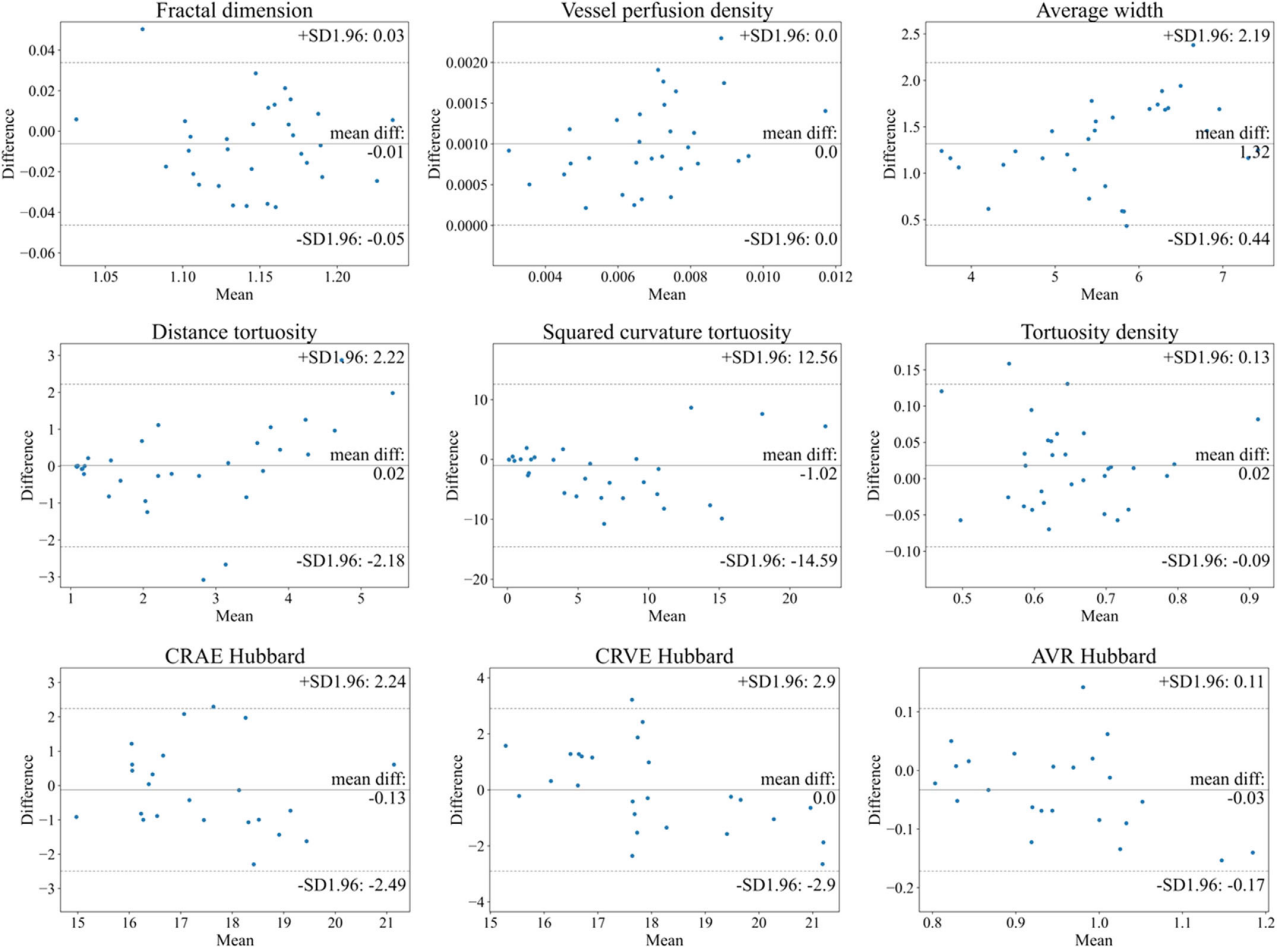
Bland–Altman plots of vascular feature agreement between expert annotation and AutoMorph segmentation at Zone B. The first two row features (e.g., tortuosity, fractal dimension) were calculated with the binary vessel segmentation map from DR HAGIS; the last row features (caliber) were measured with the artery/vein segmentation map from IOSTAR-AV. In each subplot, the *central line* indicates the mean difference and two *dashed* *lines* represent 95% limits of agreement. The unit of average width, CRAE, and CRVE is the pixel, as resolution was unknown.

The ICCs between AutoMorph features and expert features are listed in Table 3. For binary vessel morphology, the fractal dimension, vessel density, and average width metrics all achieve excellent reliability (ICC > 0.9). The other metrics show good consistency. Bland–Altman plots for Zone B are shown in Figure 6. All features show agreement. For the fractal dimension, the mean difference (MD) is –0.01, with 95% limits of agreement (LOA) of –0.05 to 0.03; for vessel density, the MD is 0.001, with 95% LOA of 0 to 0.002; for the average width, the MD is 1.32 pixels, with 95% LOA of 0.44 to 2.19; for distance tortuosity, the MD is 0.02, with 95% LOA of –2.18 to 2.22; for squared curvature tortuosity, the MD is –1.02, with 95% LOA of –14.59 to 12.56; for tortuosity density, the MD is 0.02, with 95% LOA of –0.09 to 0.13; for CRAE Hubbard, the MD is –0.13, with 95% LOA of –2.49 to 2.24; for CRVE Hubbard, the MD is 0, with 95% LOA of –2.9 to 2.9; and for AVR Hubbard, the MD is –0.03, with 95% LOA of –0.17 to 0.11. The results at Zone C and the whole image are provided in Supplementary Figures S7 and S8. Note that for the metrics CRAE, CRVE, and average width, measurements are presented in pixels, as resolution information is unknown. Some images with large errors are listed in Supplementary Figure S12.

**Table 3.** Agreement Calculation of Measured Vascular Features Between AutoMorph and Expert Annotation

	ICC (95% Confidence Interval)		
	Zone B	Zone C	Whole Image
DR HAGIS			
Fractal dimension	0.94 (0.88–0.97)	0.98 (0.95–0.99)	0.94 (0.88–0.97)
Vessel density	0.98 (0.96–0.99)	0.97 (0.94–0.99)	0.94 (0.88–0.97)
Average width	0.95 (0.89–0.98)	0.96 (0.93–0.98)	0.97 (0.95–0.99)
Distance tortuosity	0.80 (0.59–0.91)	0.85 (0.69–0.93)	0.86 (0.73–0.93)
Squared curvature tortuosity	0.68 (0.34–0.85)	0.88 (0.75–0.94)	0.84 (0.68–0.92)
Tortuosity density	0.89 (0.77–0.95)	0.70 (0.38–0.86)	0.87 (0.74–0.93)
IOSTAR-AV			
CRAE (Hubbard)	0.81 (0.56–0.92)	0.82 (0.57–0.91)	—
CRVE (Hubbard)	0.8 (0.54–0.91)	0.78 (0.52–0.89)	—
AVR (Hubbard)	0.87 (0.69–0.94)	0.81 (0.66–0.92)	—
CRAE (Knudtson)	0.76 (0.45–0.9)	0.75 (0.44–0.89)	—
CRVE (Knudtson)	0.85 (0.67–0.94)	0.86 (0.58–0.9)	—
AVR (Knudtson)	0.85 (0.66–0.94)	0.82 (0.51–0.91)	—

The agreement of vessel caliber was validated on the IOSTAR-AV dataset, other metrics with the DR HAGIS dataset. Because caliber features rely on the six largest arteries and veins in Zones B and C, there is no caliber feature for the whole image level.

### Running Efficiency and Interface

**Figure 7.**
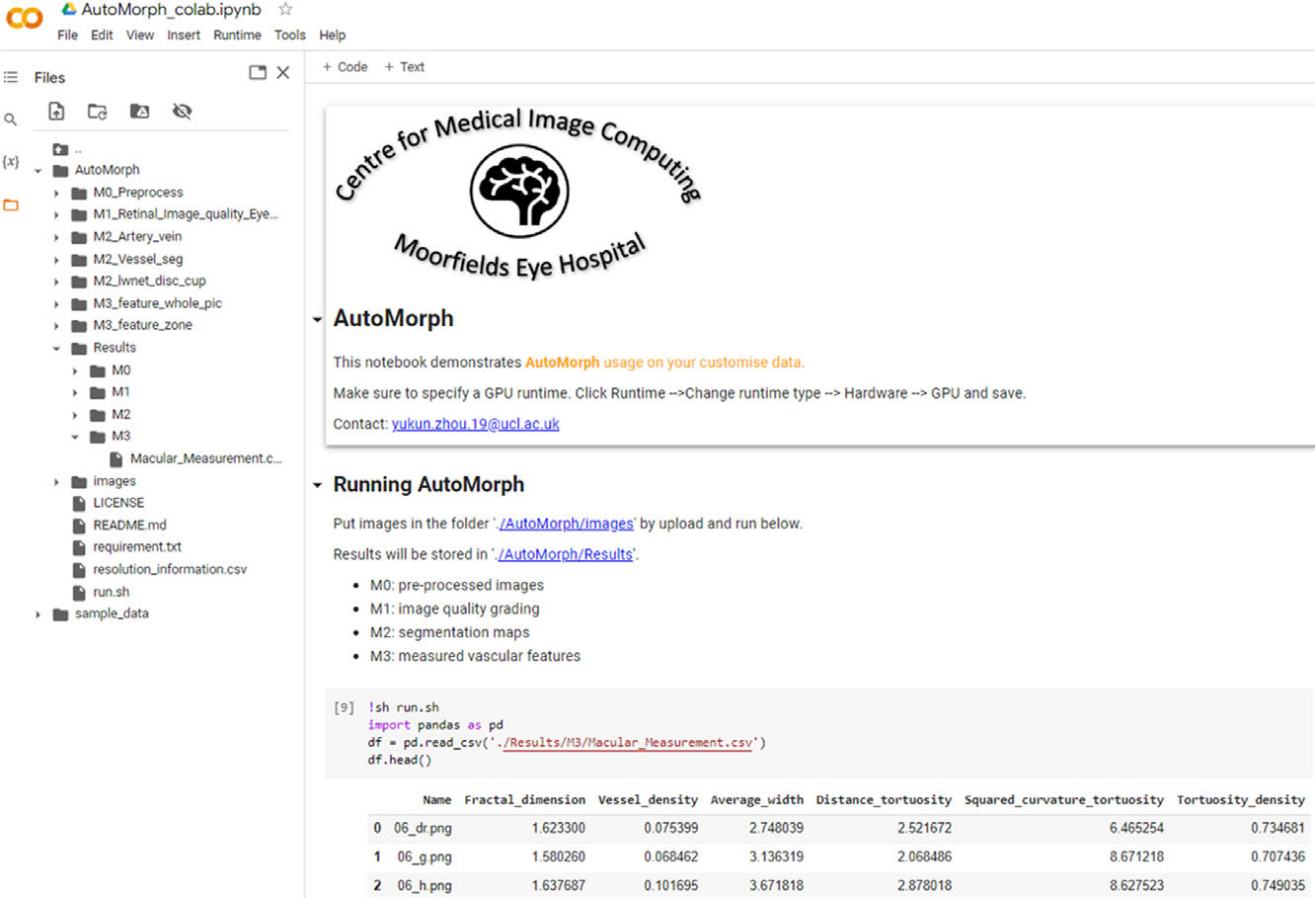
Interface of AutoMorph on Google Colaboratory. After uploading images and clicking the “run” button, all processes are executed and results stored, requiring no human intervention. The *left side* shows the files directory, and the *right bottom* lists five examples with parts of features.

The average running time for one image is about 20 seconds using a single graphics processing unit (GPU) Tesla T4 graphic card, from preprocessing to feature measurement. To ensure accessibility for researchers without coding experience, we have made AutoMorph compatible with Google Colaboratory (free GPU) (Fig. 7). The process involves placing images in a specified folder and then clicking the “run” command. All results will be stored, including segmentation maps and a file containing all measured features.

## Discussion

In this report, the four functional modules of the AutoMorph pipeline achieved comparable, or better, performance compared with the state of the art for both image quality grading and anatomical segmentation. Furthermore, our approach to confidence analysis decreased the number of false gradable images by 76%, greatly enhancing the reliability of our pipeline. Hence, we have learned that, by using a tailored combination of deep learning techniques, it is practical to accurately analyze the retinal vascular morphology in a fully automated way. Although we have evaluated the binary vessel segmentation model on the ultra-widefield retinal fundus dataset AV-WIDE, we recommend using AutoMorph on retinal fundus photographs with a 25° to 60° field of view (FOV), as all of the deep learning models are trained using images with FOVs equal to 25° to 60°, and the preprocessing step is tailored for images with this FOV.

AutoMorph maintains computation transparency despite the use of deep learning techniques. Recently, similar systems have used deep learning models to skip intermediary steps and instead directly predict morphology features. For example, the Singapore I vessel assessment (SIVA) deep learning system (DLS) predicts vessel caliber from retinal fundus images without optic disc localization or artery/vein segmentation.^3^ Another work directly predicts CVD factors from retinal fundus images in an end-to-end manner.^61^ Although these designs provide some insight into the applications of deep learning to ophthalmology, the end-to-end pipeline sacrifices transparency and raises interpretability concerns, representing a potential barrier to clinical implementation.^62^^,^^63^ Specifically, considering that some formulas are empirically defined (e.g., CRAE and CRVE are calculated based on the six widest arteries and veins), it is difficult to verify whether a model can learn this type of derivation. In contrast, the AutoMorph pipeline maintains transparency, as the individual processes can be decomposed. Models are initially employed for anatomical segmentation before vascular features are measured with traditional formulas. This process is consistent with the typical pipeline of human computation, thus improving the credibility of feature measurements.

The study cohort is selected by the image quality grading module. In this work, being different from previous work with only good-quality images, we tried to explore the effectiveness of usable images. Although purely including good-quality images can avoid potentially challenging cases for anatomic segmentation models (e.g., images with gloomy illumination), it filters out usable images that can contribute to a more general conclusion with a larger study cohort. Also, in clinical practice, a considerable number of images are usable quality but may not qualify as perfectly good quality. The pipeline developed in an environment similar to clinical reality is more prone to be deployed in the clinic. In image quality grading, the confidence analysis has recognized a considerable proportion of false gradable images and corrected them as reject quality by thresholding, as shown in Figures 3 and 4. This avoids some reject quality images failing the anatomical segmentation and then generating large errors in feature measurement. Although this thresholding increased the false ungradable cases (Fig. 4b, green box), the priority of recognizing the false gradable images is secured. Of course, it is acceptable to include only the good-quality images in the research cohorts, the same as previous work, when the quantity of good-quality images is large.

Although this work demonstrates the effectiveness of a deep learning pipeline for analyzing retinal vascular morphology, there are some challenges remaining regarding technique and standardization. First, annotating retinal image quality is subjective and lacks strict guidelines; therefore, it is difficult to benchmark external validation performance. Second, there is still room for improving anatomical segmentation, especially for artery/vein segmentation. Third, considering that the agreement varies across various vascular features (Table 3), it is necessary to compare the robustness of these features and understand the pros and cons of each one. Finally, a uniform protocol for validating retinal analysis pipelines is required, because existing software (e.g., RA^28^, IVAN,^6^ SIVA,^29^ VAMPIRE^25^) shows high variation in feature measurement.^64^^,^^65^ These four challenges exist in the field of oculomics, presenting an impediment to more extensive research.

We have made AutoMorph publicly available to benefit research in the field of oculomics, which studies the association between ocular biomarkers and systemic disease. We designed the AutoMorph interface using Google Colaboratory to facilitate its use by clinicians without coding experience. In future work, we will investigate solutions dedicated to the above challenges in oculomics research. Also, the feasibility of automatic pipeline can be extended to other modalities, such as optical coherence tomography (OCT) and OCT angiography.

## Supplementary Material

Supplement 1
