# Supplementary material for: AutoMorph: Automated Retinal Vascular Morphology Quantification Via a Deep Learning Pipeline
Source: Transl Vis Sci Technol. 2022 Jul 14;11(7):12. doi: 10.1167/tvst.11.7.12 (PMC9290317; doi:10.1167/tvst.11.7.12)
Supplement: Supplement 1 [file tvst-11-7-12_s001.docx]

**Supplementary material: Feasibility of Analysing Retinal Vascular Morphology via a Deep Learning Pipeline**

**S1. Datasets**

**Dataset and ground truth**

All the datasets for model training and external validation are publicly available. Each dataset consists of a number of retinal fundus photographs and corresponding ground truth. We summarise the characteristic of each dataset as well as the information of the ground truth.

Image quality grading datasets include EyePACS-Q and DDR-test. EyePACS-Q is based on EyePACS, which contains a large set of images collected by different imaging devices during diabetic retinopathy screening. During EyePACS-Q construction, two experts were asked to grade the image quality into three categories: good, usable, and reject. Good quality images have no low-quality factors, such as uneven illumination and blur, and all retinopathy characteristics are clearly visible. Usable quality images show some slight low-quality factors, but the optic disc, macula region, and lesions are clear enough to be identified by ophthalmologists. Reject quality has a serious quality issue and cannot be used to provide a full and reliable diagnosis, even by ophthalmologists [^31^](https://paperpile.com/c/YK7hUy/KWrs). For the DDR-test dataset, there are two categories for image quality grading, gradable and ungradable. Images with a blurring area of more than 70% and without clearly visible diabetic retinopathy lesions are considered ungradable. Seven professional graders have been trained by ophthalmologists. The seven graders vote to evaluate the images in question, and the final classification was determined by majority voting. If the seven graders could not determine a classification result, they will consult more experienced experts [^32^](https://paperpile.com/c/YK7hUy/7f8KS). The EyePACS-Q and DDR datasets indeed establish the ground truth based on the expert assessment of the image’s gradability/diagnosability. This can help identify retinal fundus photographs with serious artefacts, uneven illumination, and low contrast, which are also challenging cases for the deep learning segmentation methods (anatomical segmentation modules). In this case, by filtering the ungradable images, we can get a more accurate segmentation map as well as a precise vascular feature estimation.

For binary vessel segmentation, the ground truth map has the same size as the retinal fundus photographs. The vessel pixels are white colour and the background pixels are black. The DRIVE [^33^](https://paperpile.com/c/YK7hUy/UYggW) dataset consists of a total of 40 retinal fundus photographs, including 7 diabetic retinopathy lesions. These images were labelled by an ophthalmological expert. For the STARE [^34^](https://paperpile.com/c/YK7hUy/LITIV) dataset, ten of the images are of patients with no pathology and ten of the images contain pathology that obscures or confuses the blood vessel appearance in varying portions of the image. CHASEDB1 [^35^](https://paperpile.com/c/YK7hUy/5GEqB) is acquired from multiethnic school children, being a part of a cardiovascular health survey in 200 primary schools in London, Birmingham, and Leicester, and labelled by two experts. The ground truth of the HRF [^36^](https://paperpile.com/c/YK7hUy/B52Db) dataset is generated by a group of experts working in the field of retinal image analysis and clinicians from the cooperated ophthalmology clinics. The IOSTAR [^37^](https://paperpile.com/c/YK7hUy/C3mEV) dataset is annotated by a group of experts working in the field of retinal image analysis. The LES-AV [^38^](https://paperpile.com/c/YK7hUy/Aycuo) comprises 22 fundus photographs with available manual segmentations of the retinal vessels and their expert classification into arteries and veins. The external validation data includes DR-HAGIS [^40^](https://paperpile.com/c/YK7hUy/2o4gF) and AV-WIDE [^19,39^](https://paperpile.com/c/YK7hUy/MUBEB+nw3om). DR-HAGIS has 39 fundus images with four subgroups, glaucoma, hypertension, diabetic retinopathy, and age-related macular degeneration. The manual segmentation is provided by an expert grader. AV-WIDE provides the graph-based annotations by an expert ophthalmologist and AV-WIDE is a widefield retinal fundus dataset.

The artery/vein ground truth map is characterised by four colours: red for arteries, blue for veins, green for uncertain pixels (unidentifiable vessels at intersections of arteries and veins), and black for the background. We use three training datasets, the DRIVE-AV [^33,41^](https://paperpile.com/c/YK7hUy/UYggW+ZpU4l), LES-AV [^38^](https://paperpile.com/c/YK7hUy/Aycuo), and HRF-AV [^36,42^](https://paperpile.com/c/YK7hUy/B52Db+o7nSk), and one external validation dataset IOSTAR-AV. The DRIVE-AV dataset supplements the artery/vein ground truth by an expert to the DRIVE dataset. HRF-AV’s initial labelling was carried out by an expert in retinal image analysis, and then carefully corrected by an ophthalmologist. IOSTAR-AV [^37,43^](https://paperpile.com/c/YK7hUy/qDISc+C3mEV) is annotated by a group of experts working in the field of retinal image analysis.

Optic disc segmentation is a binary segmentation task that classifies each pixel as the optic disc pixel or the background. The ground truth includes the white colour pixels for the optic disc and black colour for the background. For the REFUGE dataset, the ground truths of the optic disc were provided by seven independent glaucoma specialists from the Zhongshan Ophthalmic Center (Sun Yat-sen University, China). All the ophthalmologists independently reviewed and a single segmentation per image was afterwards obtained by taking the majority voting of the annotations of the seven experts. A senior specialist with more than 10 years of experience in glaucoma performed a quality check afterwards, analyzing the resulting masks to account for potential mistakes [^44^](https://paperpile.com/c/YK7hUy/anU6Z). For the GAMMA dataset, four clinical ophthalmologists manually annotated the initial segmentation region of the optic disc for each fundus image. The senior ophthalmologist then fused the results of the four initial segmentation results and selected the intersection of the annotated results of several ophthalmologists as the final ground truth [^46^](https://paperpile.com/c/YK7hUy/ylFl)^,^[^45^](https://paperpile.com/c/YK7hUy/l8Bd6). For the external validation dataset IDRID, all observers were trained by expert ophthalmologists for the identification of individual lesions and optic discs. Later the markings on each of these images were reviewed by two retinal specialists, and they were finalized when the necessary consensus was reached [^47^](https://paperpile.com/c/YK7hUy/6rMVT).

Some examples are shown in Figure S1 to depict the visualisation difference brought by different imaging devices and research studies.


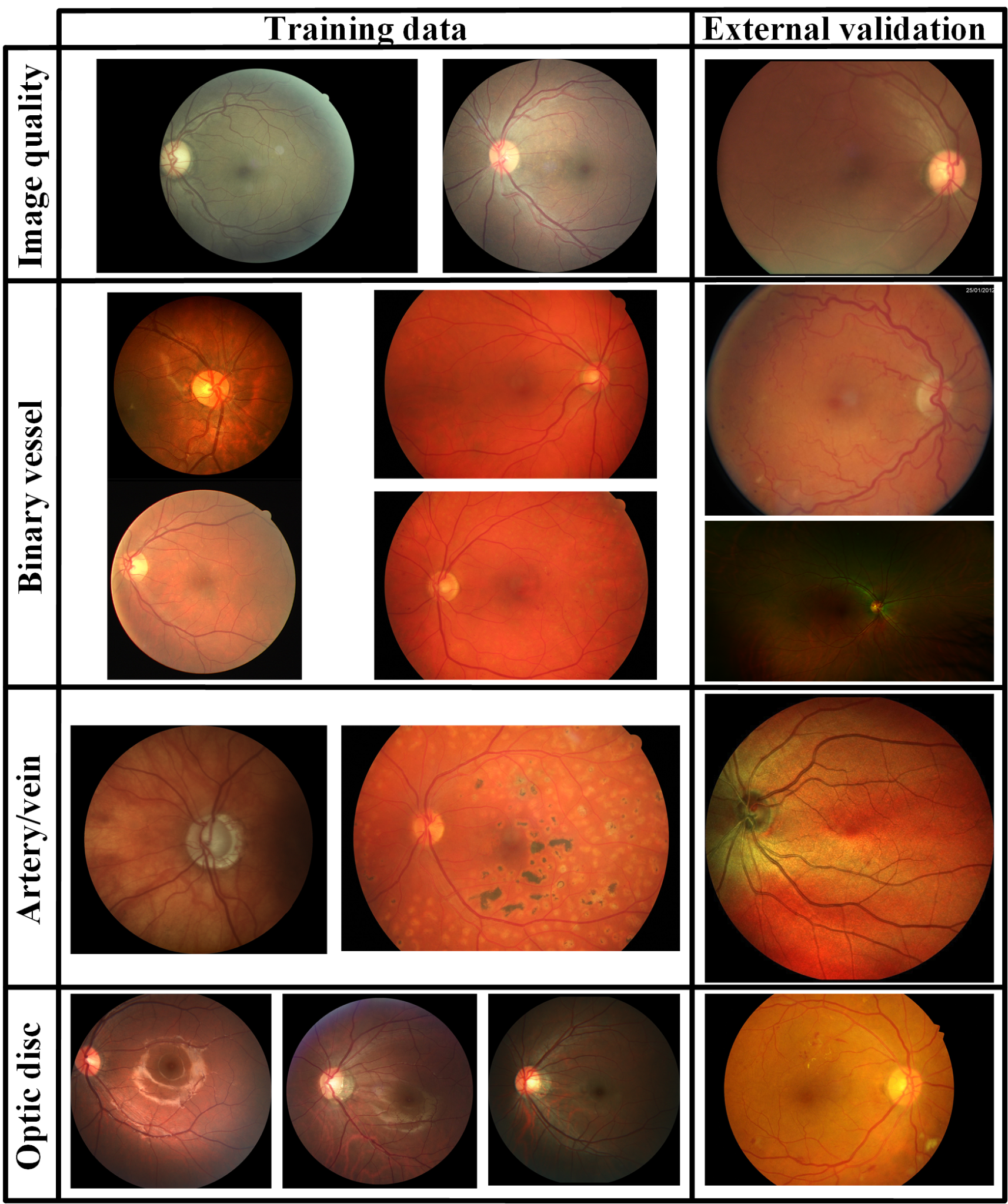


Figure S1. Image examples of training data and external validation data for the image quality grading, binary vessel segmentation, artery/vein segmentation, and optic disc segmentation.

**S2. Image quality grading module**

**Image pre-processing**

The images are squared as Figure S2.


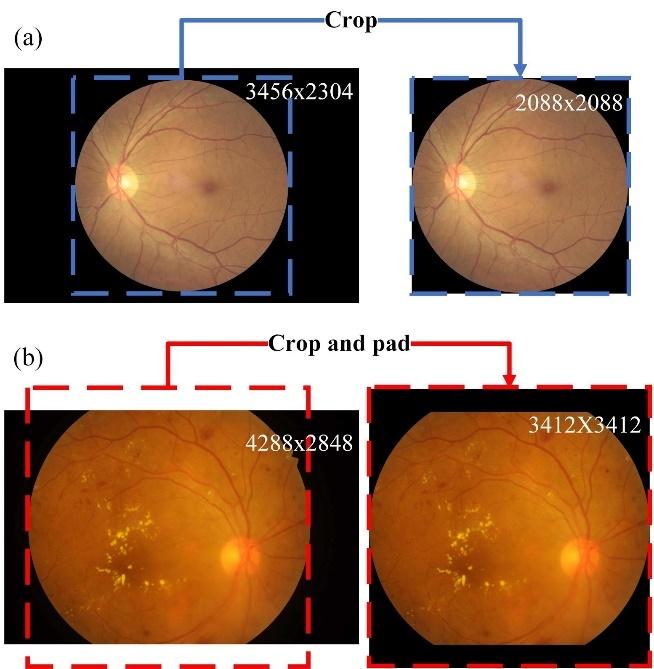


Figure S2. Two examples of retinal fundus image pre-processing. The redundant background is removed by cropping in (a) and (b), whilst some padding is employed in (b) to make the image conform to a geometric shape. The image size is listed at the top right corner.

**Model structure selection**

We compared the classification performance with different classification backbones, namely ResNeXt101-32x8d, EfficientNet-B4, and EfficientNet-B5. The models are pretrained on the ImageNet dataset, and the final classifier neurons are substituted from 1000 to 3, respectively corresponding to the category of good, usable, and reject image quality. The training epoch is 30 and batch size is 8. The initial learning rate is 0.0002 and the optimiser is Adam. The learning rate schedule and early stopping are employed to avoid model overfitting. The cross entropy loss function is used and the checkpoint with lowest loss is stored for inference. We use four Tesla T4 GPUs to train the model. As shown in Figure S3, the performance of the three backbones shows no statistical difference (p>0.05). In this case, we use the EfficientNet-b4 as the backbone considering its lower parameters compared with EfficientNet-b5 and ResNeXt101-32x8d.


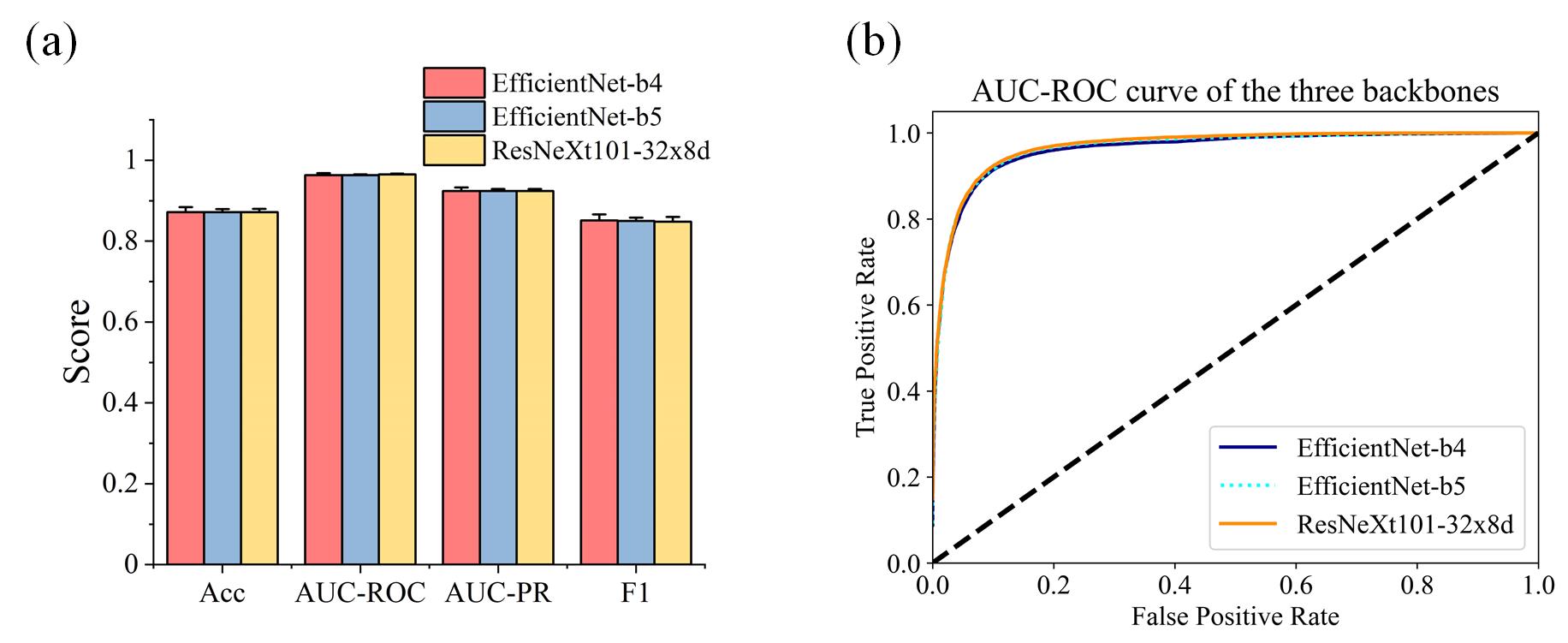


Figure S3. Performance comparison of the quality grading models with different backbones. (a) verifies there is no significant difference between the three backbones and (b) shows that the AUC-ROC curves highly overlap. For each backbone, eight models are trained on different subsets of EyePACS-Q training data, and then validated on EyePACS-Q validation data. Acc, accuracy. AUC-ROC, Area-under-curve Receiver operating characteristic. AUC-PR, Area-under-curve Precision-Recall. F1, F1-score.

**Confidence analysis threshold**

We calculate the mean value and SD of ensemble probability in confidence analysis. Depicted in Figure S4, comparing the distribution of true gradable and false gradable in the tuning data (20% of training data used to tune the training hyperparameters during training), we found the FP cases have lower mean value and higher SD. Threshold choices were made by inspecting average probability histograms and standard deviation histograms. We selected thresholds that resulted in a desirable trade-off, i.e., reduced a large proportion of false gradables whilst introducing an acceptable number of false ungradables. The average threshold of 0.75 and SD threshold of 0.1 are employed.


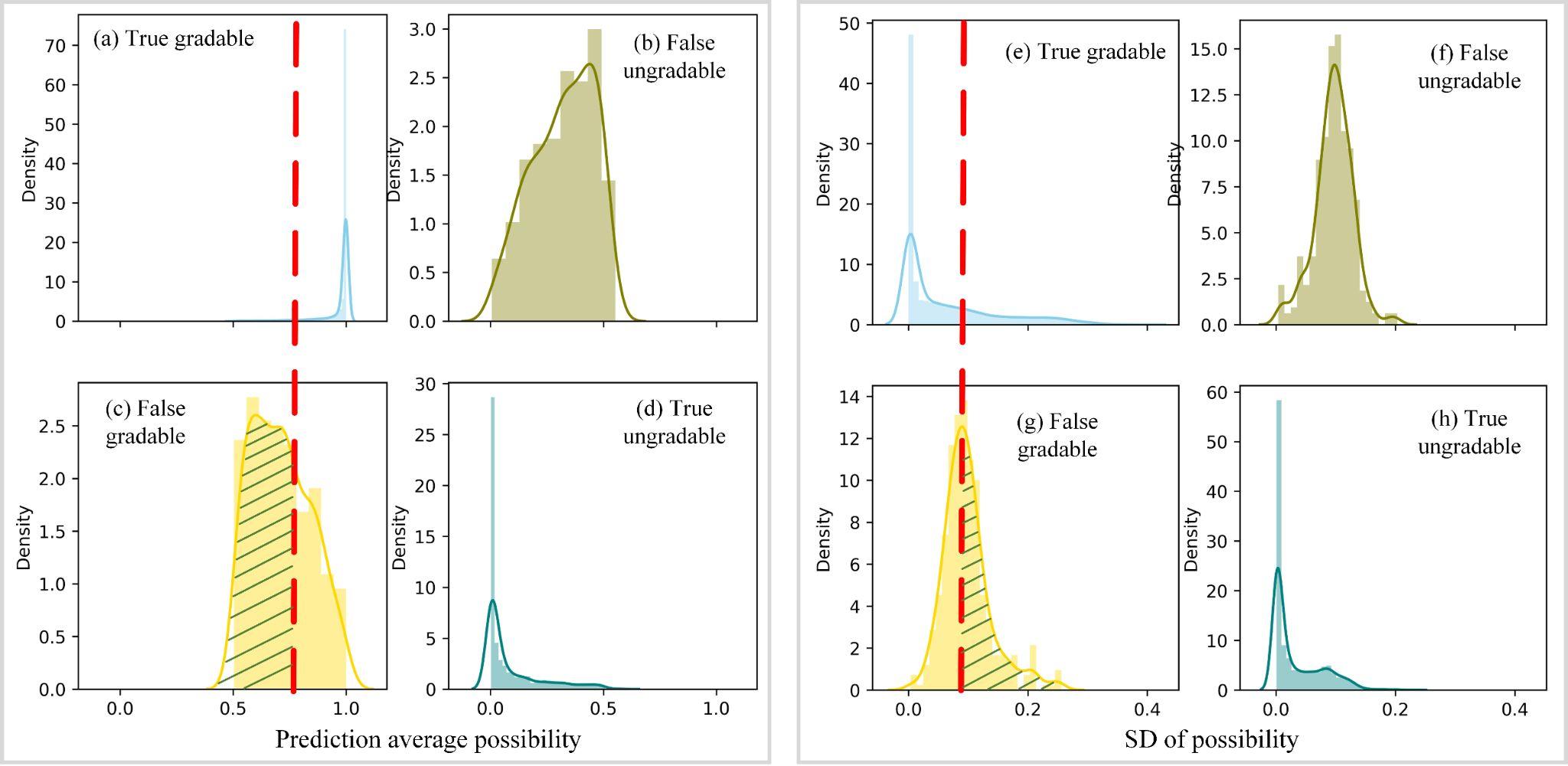


Figure S4. (a-d) show the average probability histogram, respectively indicating true gradable, false ungradable, false gradable, and true ungradable. (e-h) are corresponding SD histograms. Comparing the true gradable and false gradable, it’s observed that the average probability of true gradable (a) concentrates to 1 and SD (e) is closer to 0. By thresholding on an average probability 0.75 and SD threshold 0.1 (red dash line), lots of false gradable images can be rectified as the ungradable images and then being filtered out, depicted in regions with green slash lines.

**Confusion matrix on DDR**

The confusion matrix and AUC-ROC curve on DDR are listed in Figure S5.


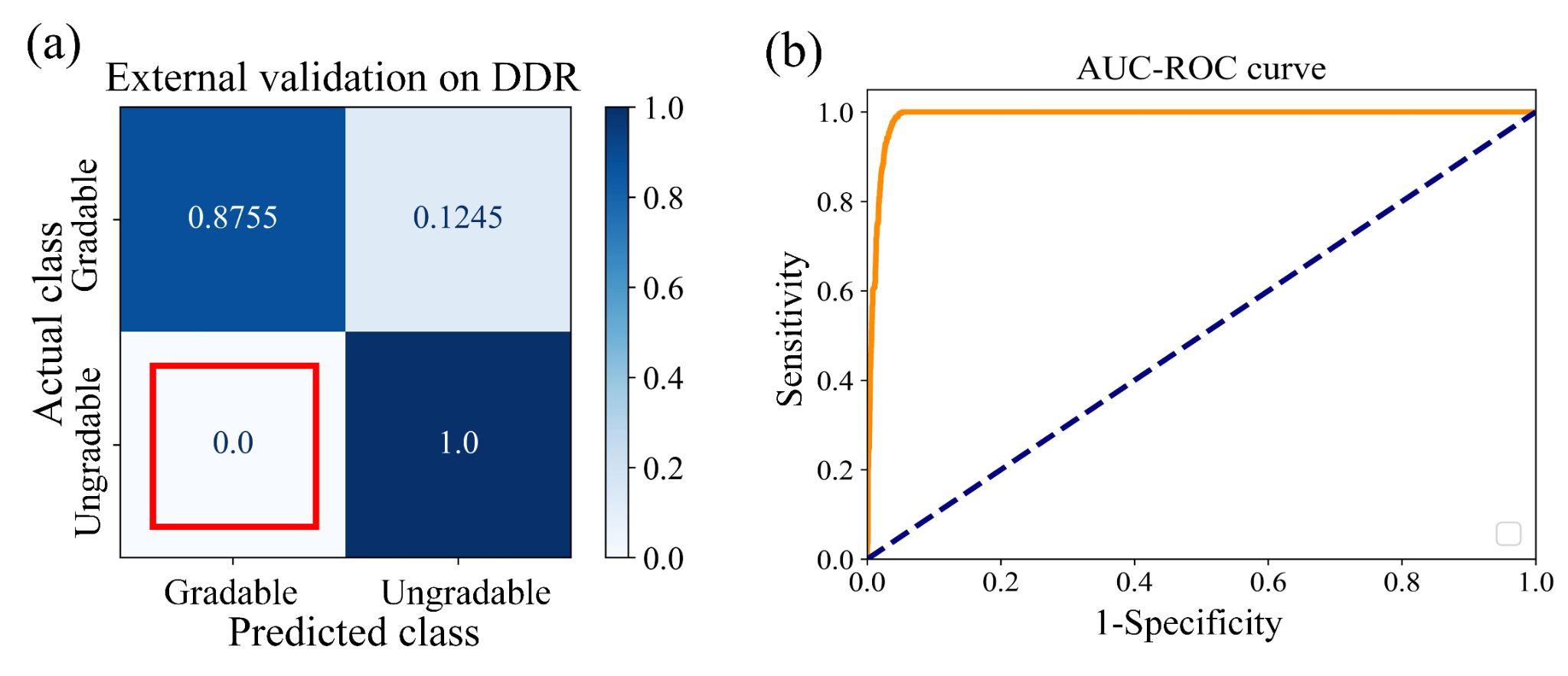


Figure S5. The confusion matrix (a) and AUC-ROC curve (b) showing the performance of AutoMorph on the external DDR test. The ratio of FP-gradable is 0 which says all ungradable images are correctly recognised, thus remaining no false gradable images for the following anatomical segmentation steps. The AUC-ROC curve indicates good performance of AutoMorph on the DDR test data.

**S3. Anatomical segmentation modules**

**Binary vessel segmentation:** SEGAN is a variant of U-Net with enhanced performance in multi-scale information learning. It consists of a segmenter and a discriminator, which are trained in adversarial learning strategy. The input of the network is the retinal fundus photographs and the output is a segmentation map with the same size of the input. The pre-processed images are resized to (912, 912) to ease the computational stress. The segmentation map will be resized to the original size for feature measurement. Each pixel of the segmentation map shows the probability of being vessel. After thresholding with 0.5, the pixels with values of 1 are vessels. The training epoch is 600 and batch size is 2. The initial learning rate is 0.0002 and the optimiser is Adam. The learning rate schedule is employed for robust convergence. Three loss functions are utilised, namely adversarial loss, mean square error, and cross entropy loss. The checkpoint with the highest F1-score is stored. We use one Tesla T4 GPUs to train the model.

**Artery/vein segmentation:** BFN decomposes the multi-class task into multi binary tasks, followed by a binary-to-multi-class information fusion, so as to correct the information around the intersections. Different segmenters share the same discriminator. The input of the network is the retinal fundus photographs and the output is a multi-class segmentation map with the same size of the input. The pre-processed images are resized to (720, 720) to ease the computational stress. The multi-class segmentation map will be resized to the original size for feature measurement. Each pixel of the segmentation map shows the four-element probability of being artery, vein, uncertain pixels, and background. After selecting the maximised probability as the final category, the pixels with values of 0 are background, 1 for arteries, 2 for veins, and 3 for uncertain pixels. The red, blue, green, and black colours are transformed visualisation. The training epoch is 1500 and batch size is 2. The initial learning rate is 0.0008 and the optimiser is Adam. The learning rate schedule is employed. Three loss functions are utilised, namely adversarial loss, mean square error, and cross entropy loss. The checkpoint with the highest F1-score is stored. One Tesla T4 GPU is employed to train the model.

**Optic disc segmentation:** LW-Net consists of two simplified U-Net, where the first part generates a coarse segmentation result and the second part polishes the segmentation detail. This cooperated regularisation between the two parts enhances model segmentation performance when pathology is present. The overall model is light-weight in terms of parameter numbers. The input of the network is the retinal fundus photographs and the output is a segmentation map with the same size of the input. The pre-processed images are resized to (512, 512) to enable a large batch size. The segmentation map will be resized to the original size for feature measurement and the definition of the pre-defined zones. Each pixel of the segmentation map shows the probability of being optic disc. After thresholding with 0.5, the pixels with values of 1 are optic disc. The training epoch is 1000 and batch size is 16. The initial learning rate is 0.01 and the optimiser is Adam. The learning rate schedule is employed. The cross entropy loss is used and the checkpoint with the highest AUC-ROC is stored. One Tesla T4 GPU is employed to train the model.


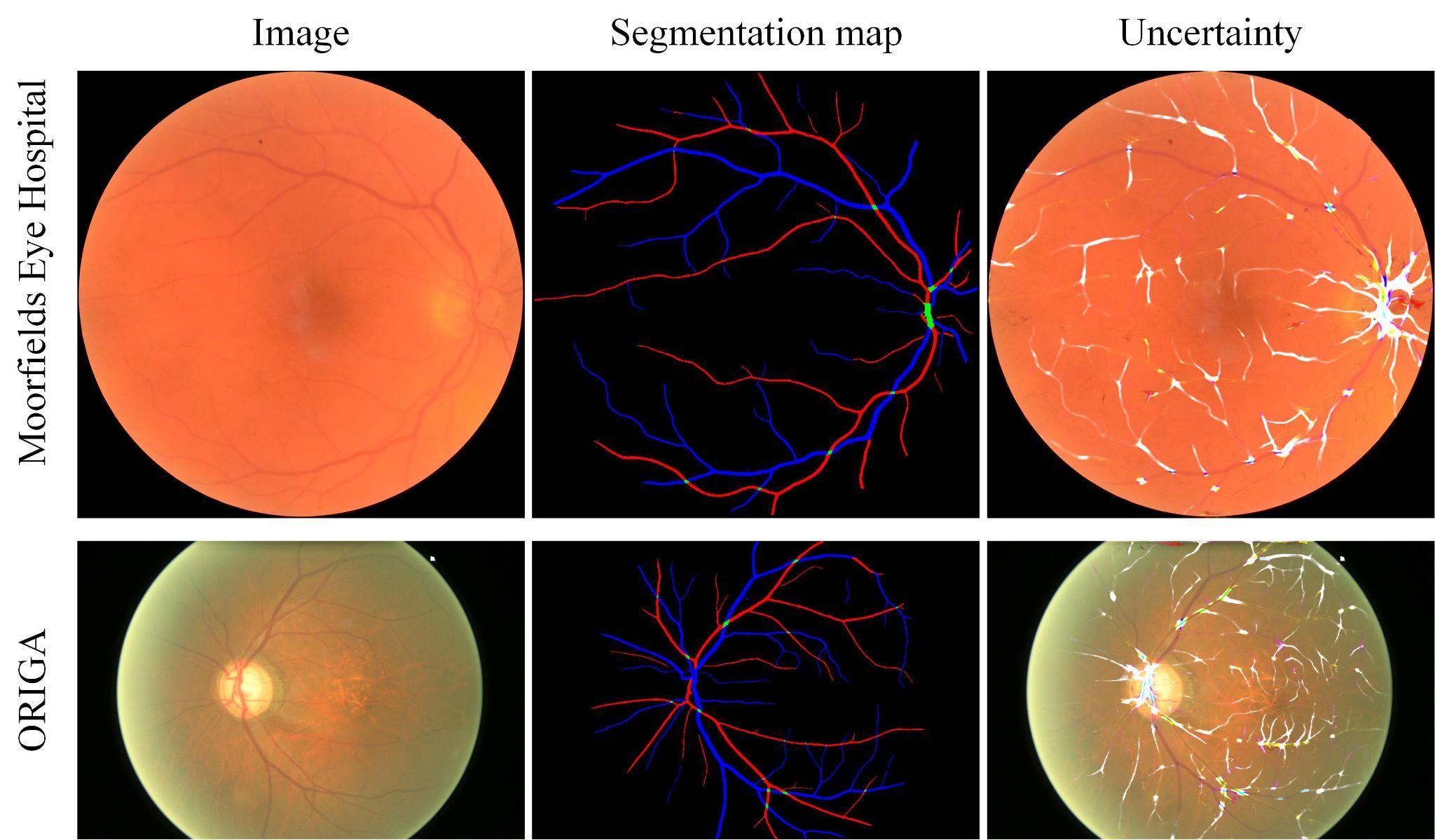


Figure S6. Artery/vein segmentation visualisation examples of challenging cases (blur and poor illumination) from Moorfields Eye Hospital and ORIGA dataset. The uncertainty map is overlaid on the retinal fundus image. Brighter white indicates higher uncertainty.

In order to give a direct technical comparison between our segmentation methods and those compared methods, we have listed the technically fair comparison in Table S1. All models are trained on the same training dataset and internally validated. As we don’t have multiple groups of results for the compared methods [^39,57–59^](https://paperpile.com/c/YK7hUy/nw3om+0HSfk+Wv42n+q41W6), we cannot test if a statistically significant difference exists. But our results show clearly better performance. We compare all the metrics they have provided. We follow the same splitting of training and testing data of the compared methods.

Table S1. Internal validation of AutoMorph and comparison to competitive methods. All models are trained and validated on the same data to compare the technical superiority.

Internal validation on **DR-HAGIS** (binary vessel segmentation)

|  | Sensitivity | Specificity | F1-score | Accuracy |
| --- | --- | --- | --- | --- |
| AutoMorph | 0.74±0.02 | 0.99±0.01 | 0.77±0.01 | 0.97±0.01 |
| Compared [^57^](https://paperpile.com/c/YK7hUy/0HSfk) | 0.67 | 0.98 | 0.71 | 0.97 |

Internal validation on **WIDE** (binary vessel segmentation)

|  | Sensitivity | Precision | F1-score | Accuracy |
| --- | --- | --- | --- | --- |
| AutoMorph | 0.81±0.02 | 0.85±0.02 | 0.83±0.02 | 0.98±0.01 |
| Compared [^39,57^](https://paperpile.com/c/YK7hUy/0HSfk+nw3om) | 0.78 | 0.82 | 0.8 | 0.97 |

Internal validation on **IOSTAR-AV** (artery/vein segmentation)

|  | Sensitivity | Specificity | Accuracy |
| --- | --- | --- | --- |
| AutoMorph | 0.74±0.04 | 0.98±0.01 | 0.97±0.02 |
| Compared [^58^](https://paperpile.com/c/YK7hUy/Wv42n) | 0.79 | 0.76 | 0.78 |

Internal validation on **IDRID** (optic disc segmentation)

|  | Sensitivity | Accuracy | IoU |
| --- | --- | --- | --- |
| AutoMorph | 0.95±0.03 | 0.99±0.01 | 0.93±0.02 |
| Compared [^58,59^](https://paperpile.com/c/YK7hUy/Wv42n+q41W6) | 0.9 | 0.99 | 0.85 |

**S4. Vascular feature in ZONE C and whole image**

Bland-Altman plots for parts of vascular morphology features at ZONE B and whole image.


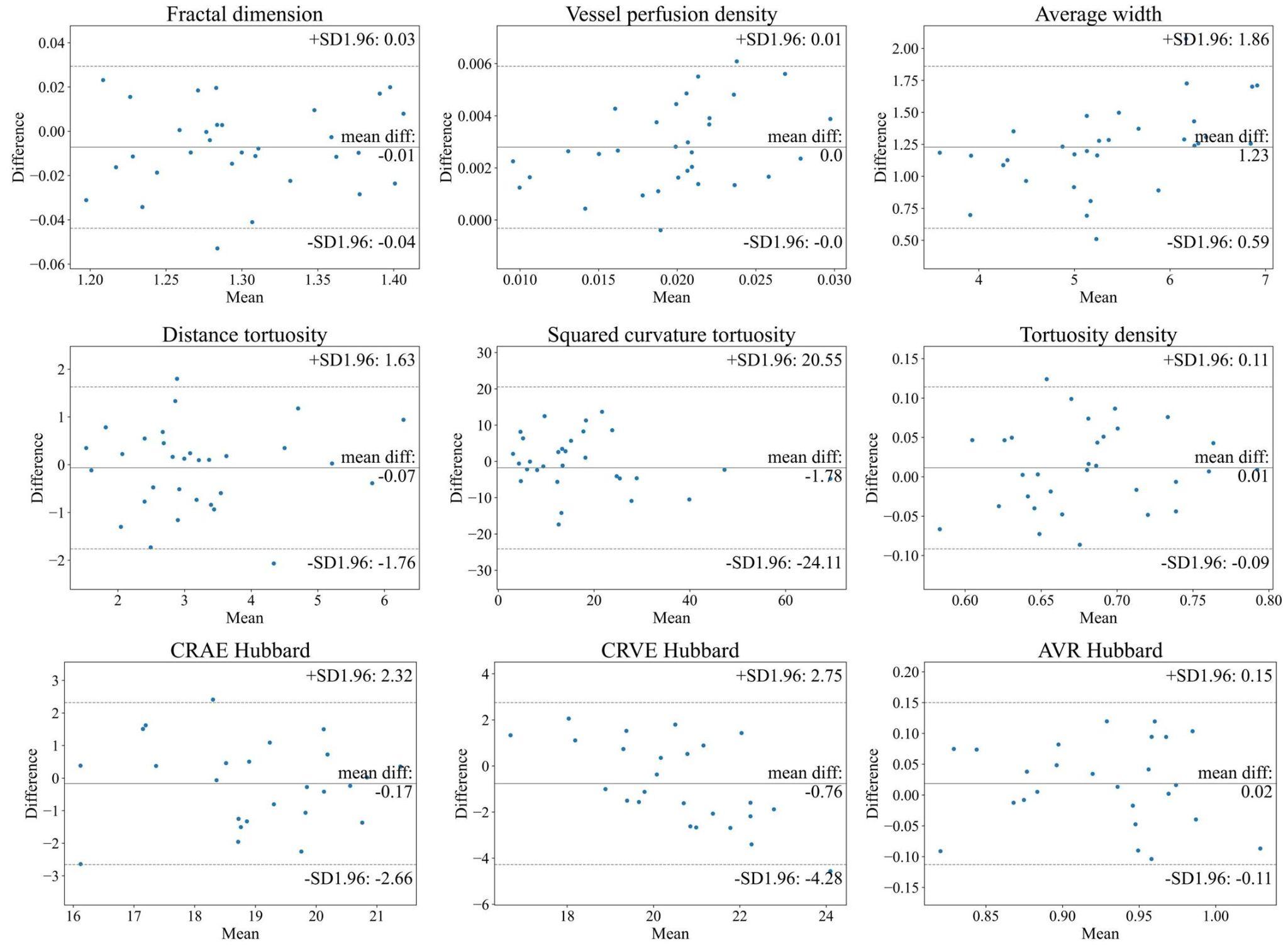


Figure S7. Bland-Altman plots of vascular feature agreement between expert annotation segmentation map and AutoMorph segmentation at ZONE C. The first two rows features (tortuosity, fractal dimension, etc.) are calculated with binary vessel segmentation map from DR-HAGIS, while the last row features (calibre) are measured with artery/vein segmentation map from IOSTAR-AV. In each subplot, the central line indicates the mean difference and two dash lines represent 95% limits of agreement.


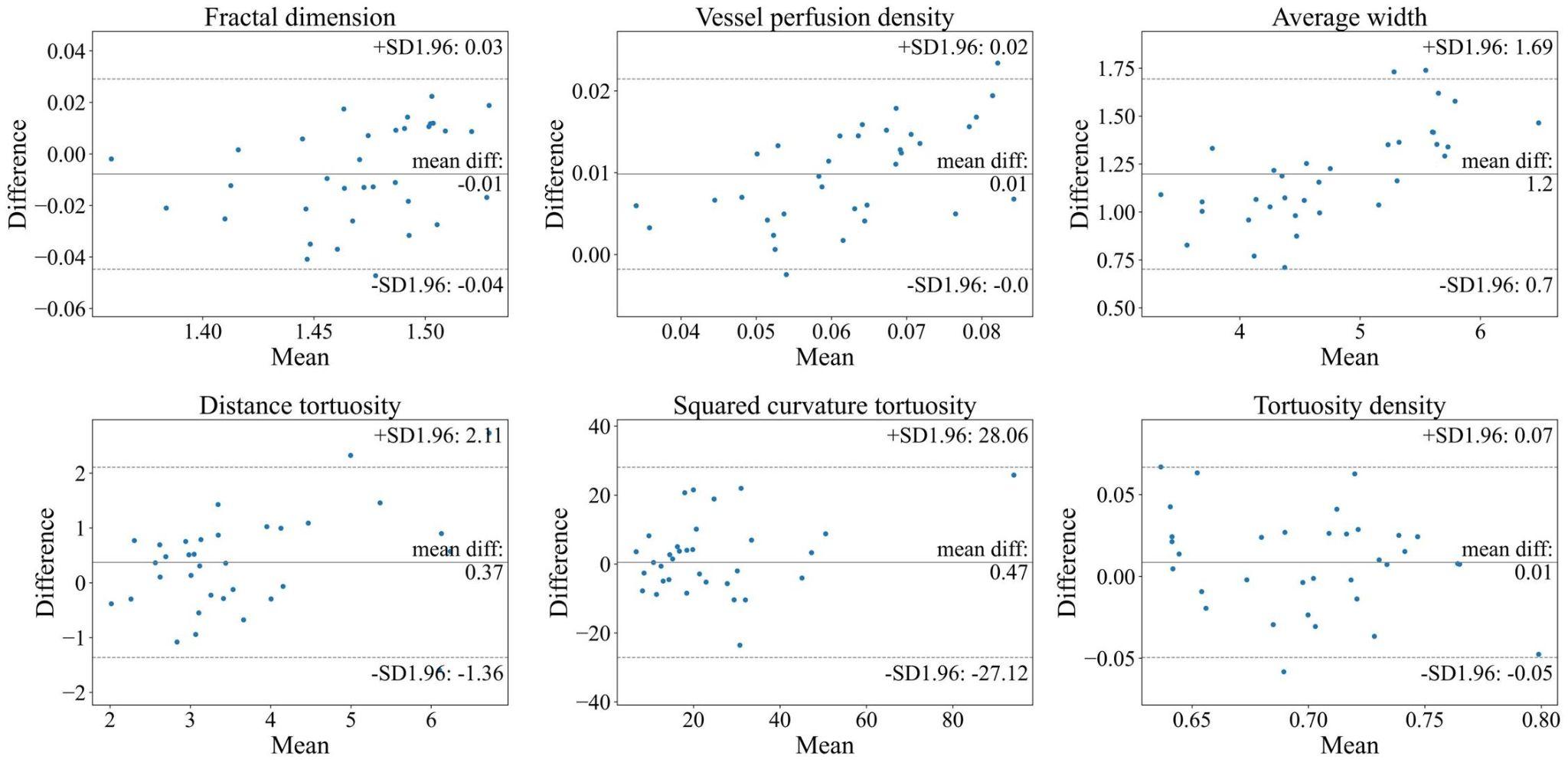


Figure S8. Bland-Altman plots of vascular feature agreement between expert annotation segmentation map and AutoMorph segmentation at the whole image.


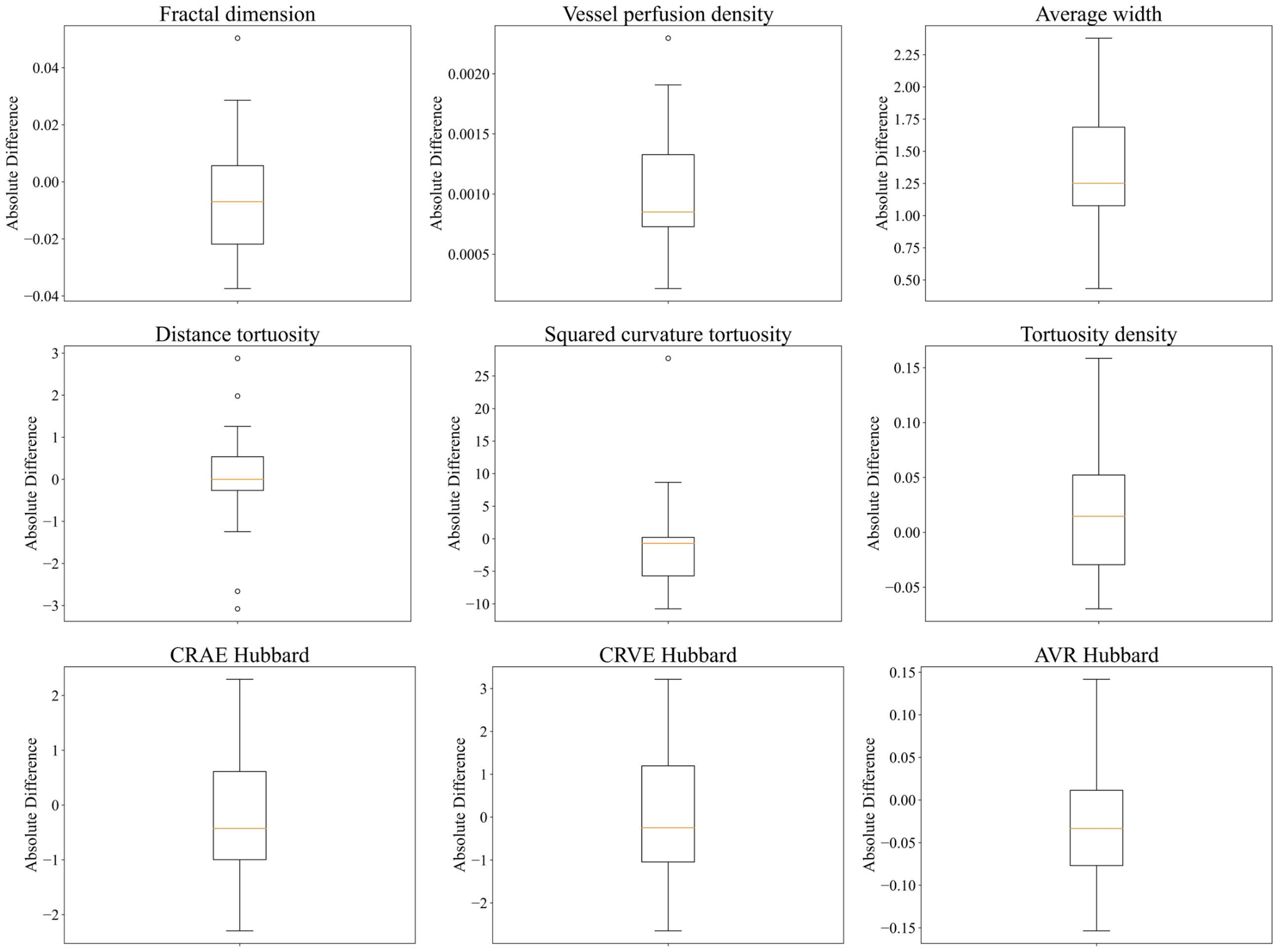


Figure S9. Boxplot of vascular feature difference between expert annotation segmentation map and AutoMorph segmentation at the ZONE B. The first two rows features (tortuosity, fractal dimension, etc.) are calculated with binary vessel segmentation map from DR-HAGIS, while the last row features (calibre) are measured with artery/vein segmentation map from IOSTAR-AV. In each subplot, the orange line indicates the mean difference and the box includes data distribution between 25 percentile to 75 percentile. Circles are outliers.


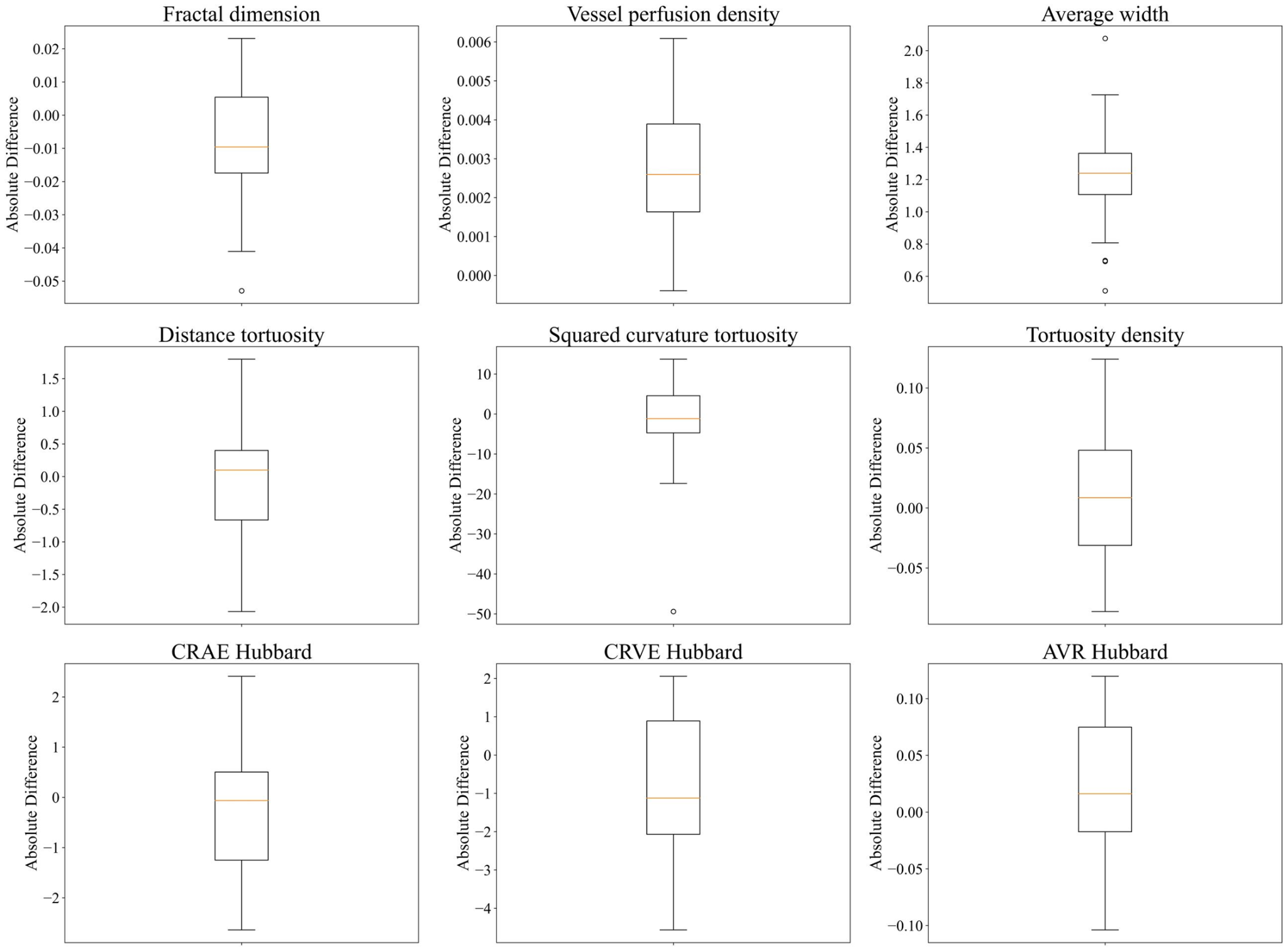


Figure S10. Boxplot of vascular feature difference between expert annotation segmentation map and AutoMorph segmentation at the ZONE C.


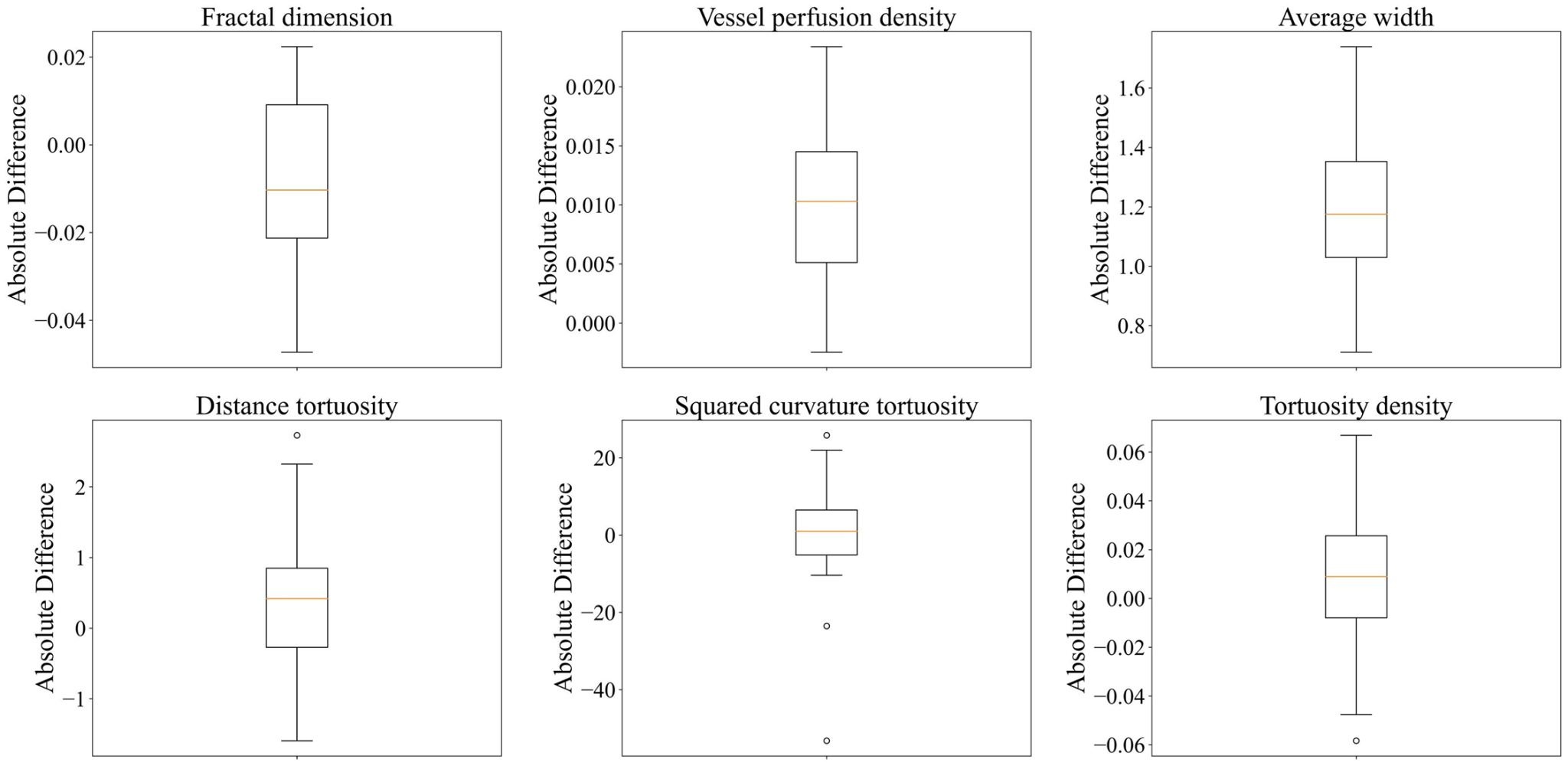


Figure S11. Boxplot of vascular feature difference between expert annotation segmentation map and AutoMorph segmentation at the whole image.


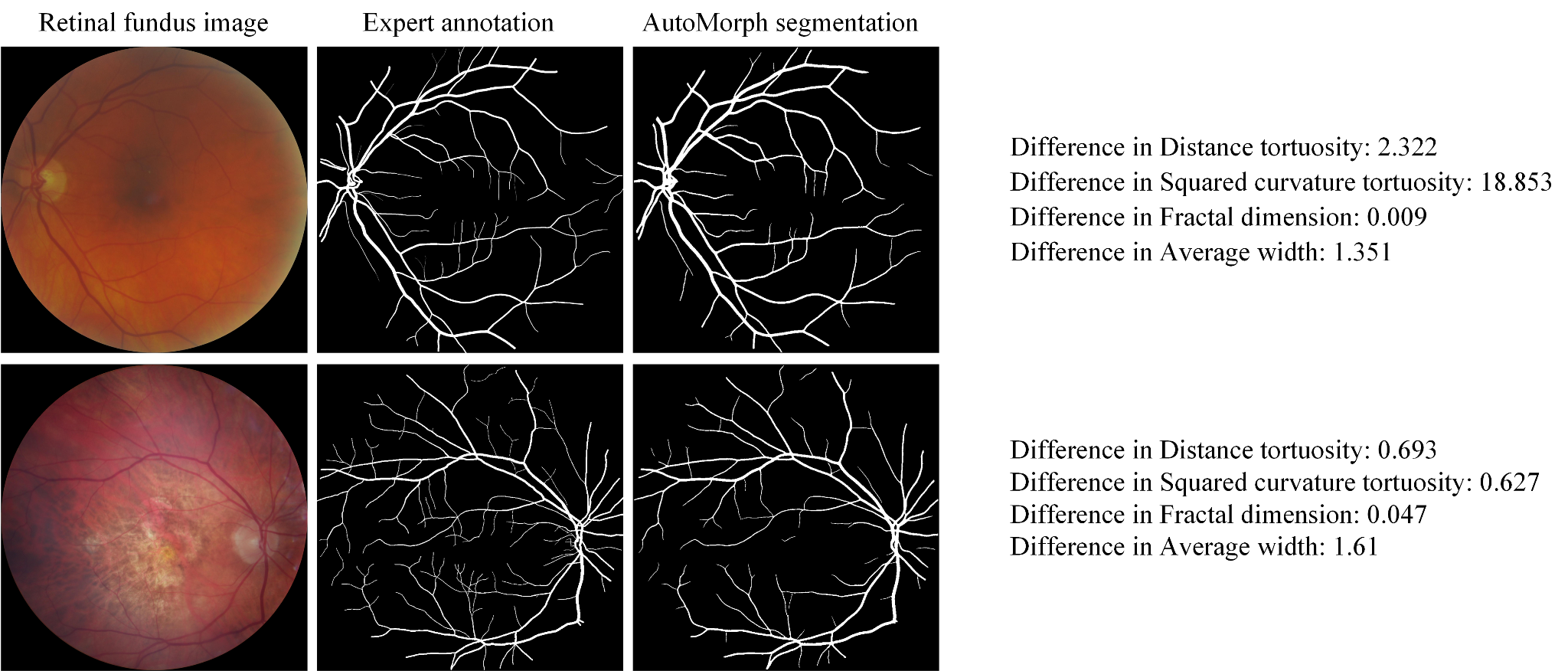


Figure S12. Examples with large vascular features’ error. Comparing the two cases, we can understand that different vascular features are sensitive to specific kinds of segmentation error according to the calculation process. Distance tortuosity and squared curvature tortuosity usually show large errors when vessels show extra junctions or miss junctions, while the fractal dimension shows errors when overall vasculature shape is considerably different to expert annotation (e.g., missing some distal vessels).

**S5. Detailed list of measured features**

All measured features are listed in Figure S13.


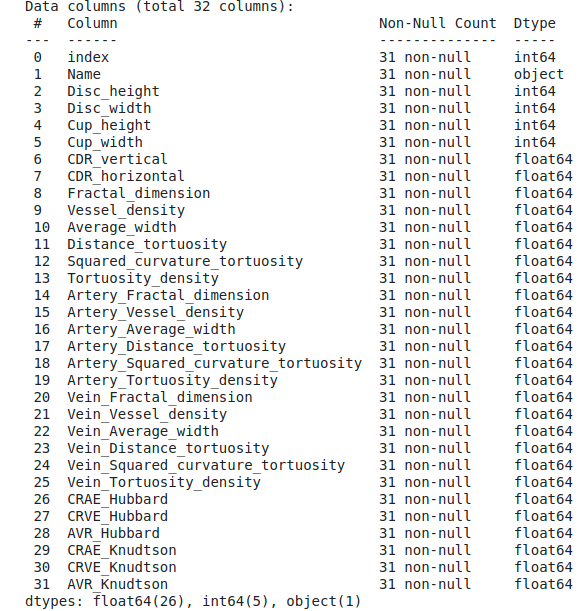


Figure S13. This is a comprehensive list for measured vascular features.
